# Supplementary material for: ER Stress and Autophagic Perturbations Lead to Elevated Extracellular α-Synuclein in GBA-N370S Parkinson's iPSC-Derived Dopamine Neurons
Source: Stem Cell Reports. 2016 Feb 18;6(3):342–56. doi: 10.1016/j.stemcr.2016.01.013 (PMC4788783; doi:10.1016/j.stemcr.2016.01.013)
Supplement: Document S1. Supplemental Experimental Procedures, Figures S1–S7, and Table S1 [file mmc1.pdf]

Stem Cell Reports, Volume *6*

Supplemental Information

ER Stress and Autophagic Perturbations Lead to Elevated Extracellular α-Synuclein in *GBA-N370S* Parkinson's iPSC-Derived Dopamine Neurons

Hugo J.R. Fernandes, Elizabeth M. Hartfield, Helen C. Christian, Evangelia Emmanoulidou, Ying Zheng, Heather Booth, Helle Bogetofte, Charmaine Lang, Brent J. Ryan, S. Pablo Sardi, Jennifer Badger, Jane Vowles, Samuel Evetts, George K. Tofaris, Kostas Vekrellis, Kevin Talbot, Michele T. Hu, William James, Sally A. Cowley, and Richard Wade-Martins

Figure-S1 (Wade-Martins)

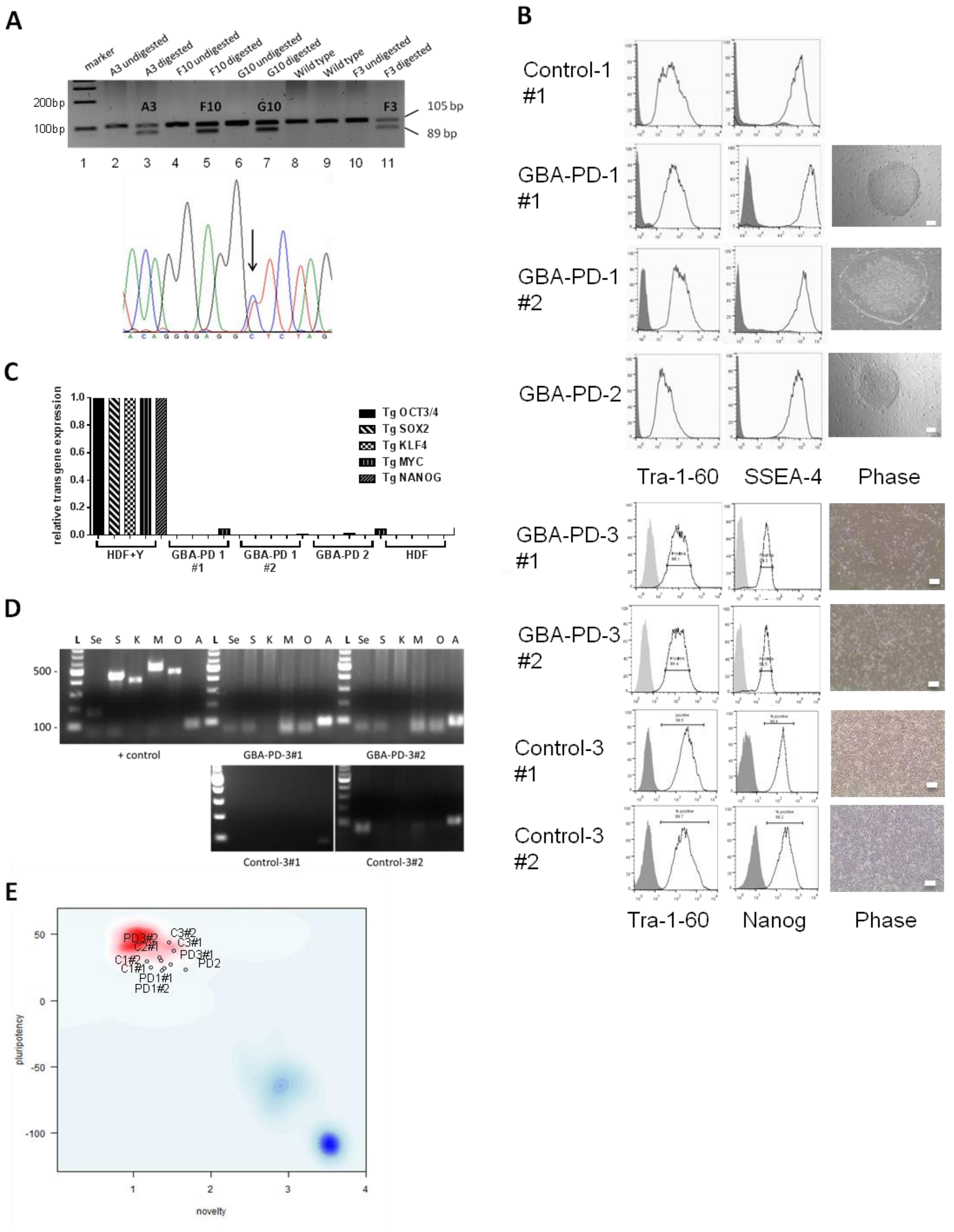

Figure-S2 (Wade-Martins)

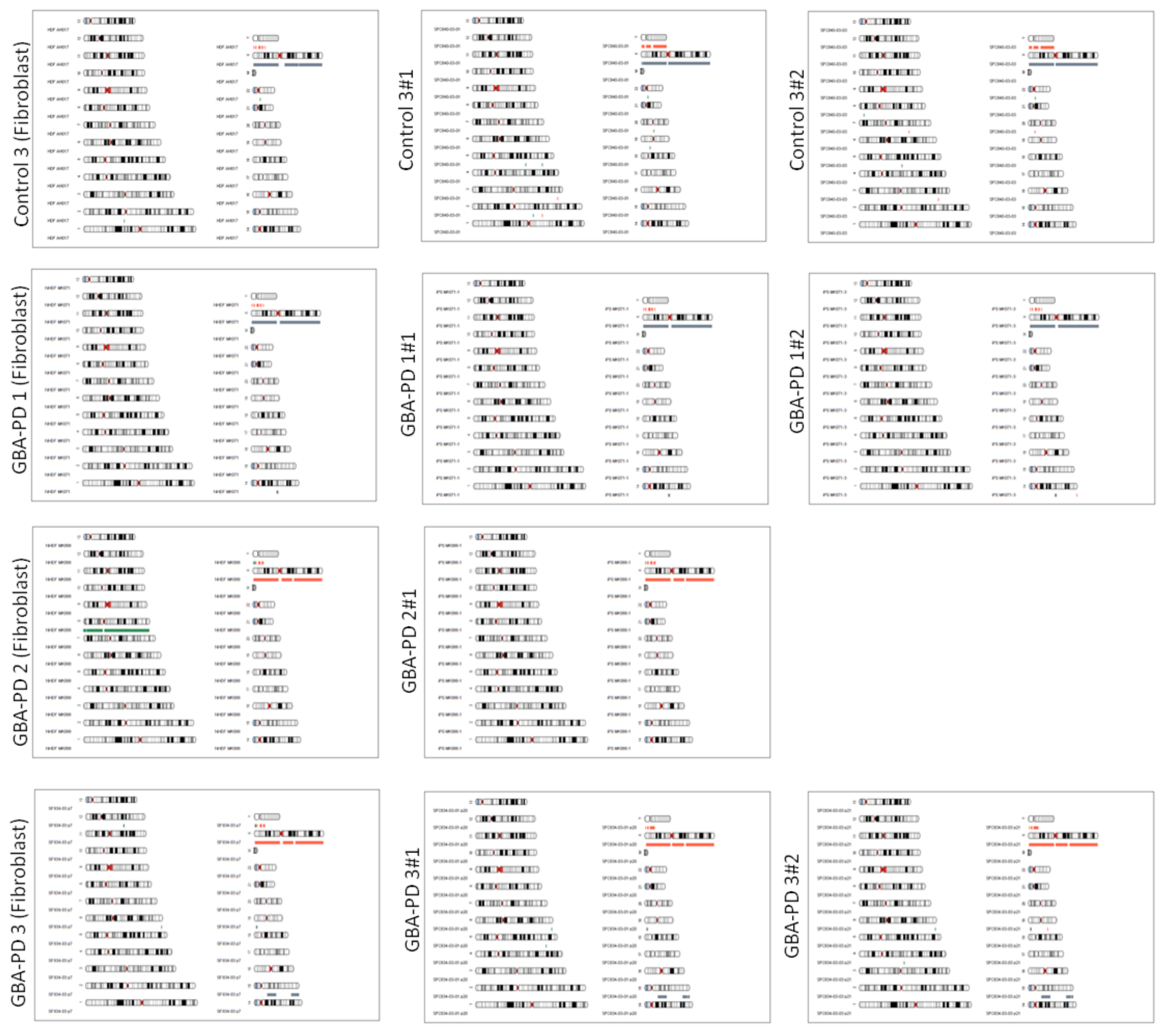

Figure-S3 (Wade-Martins)

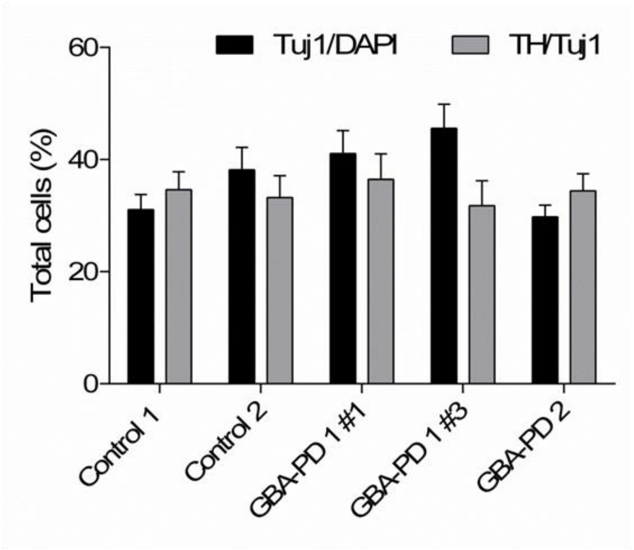

Figure-S4 (Wade-Martins)

A

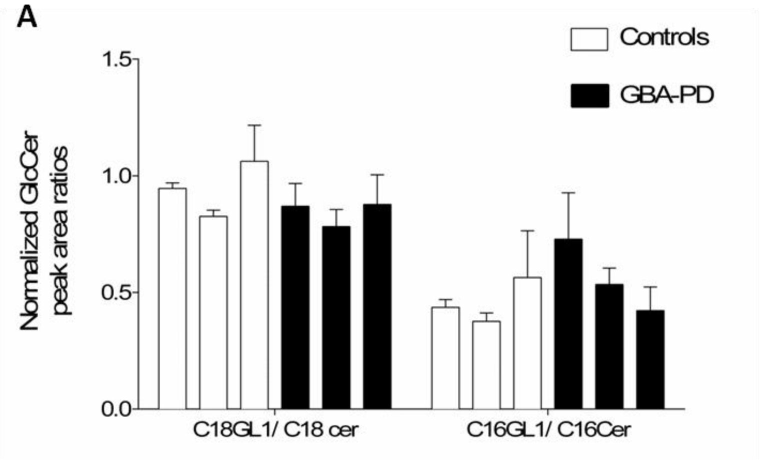

B

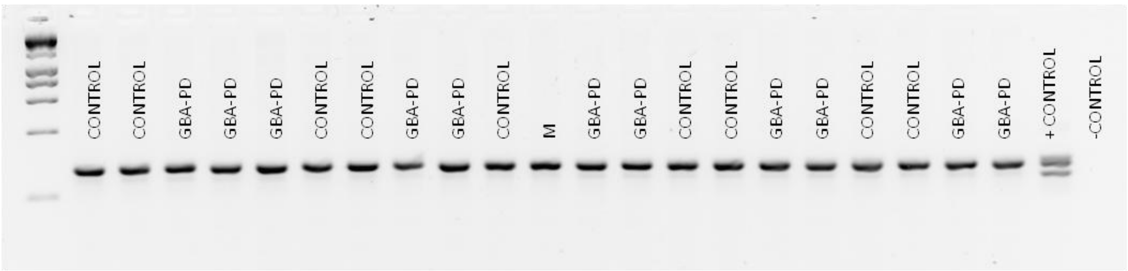

Figure-S5 (Wade-Martins)

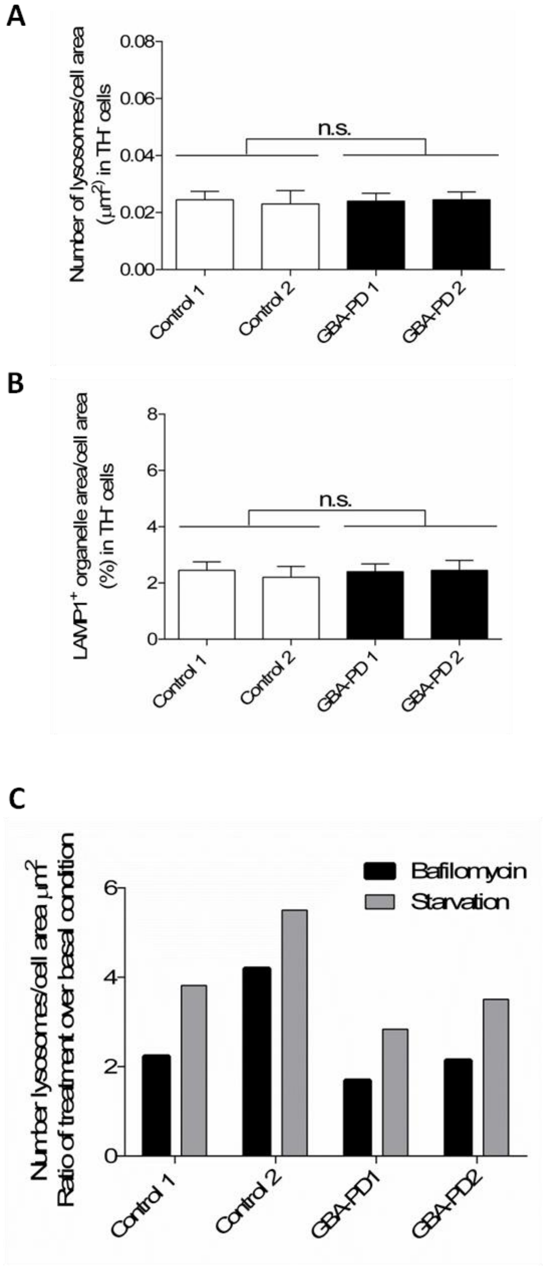

Figure-S6 (Wade-Martins)

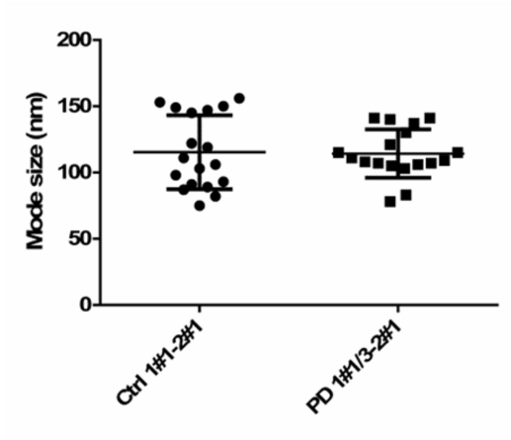

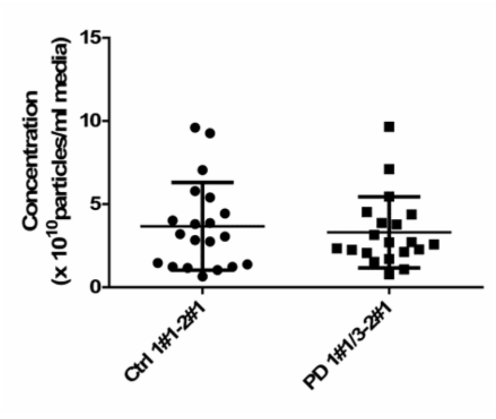

Figure-S7 (Wade-Martins)

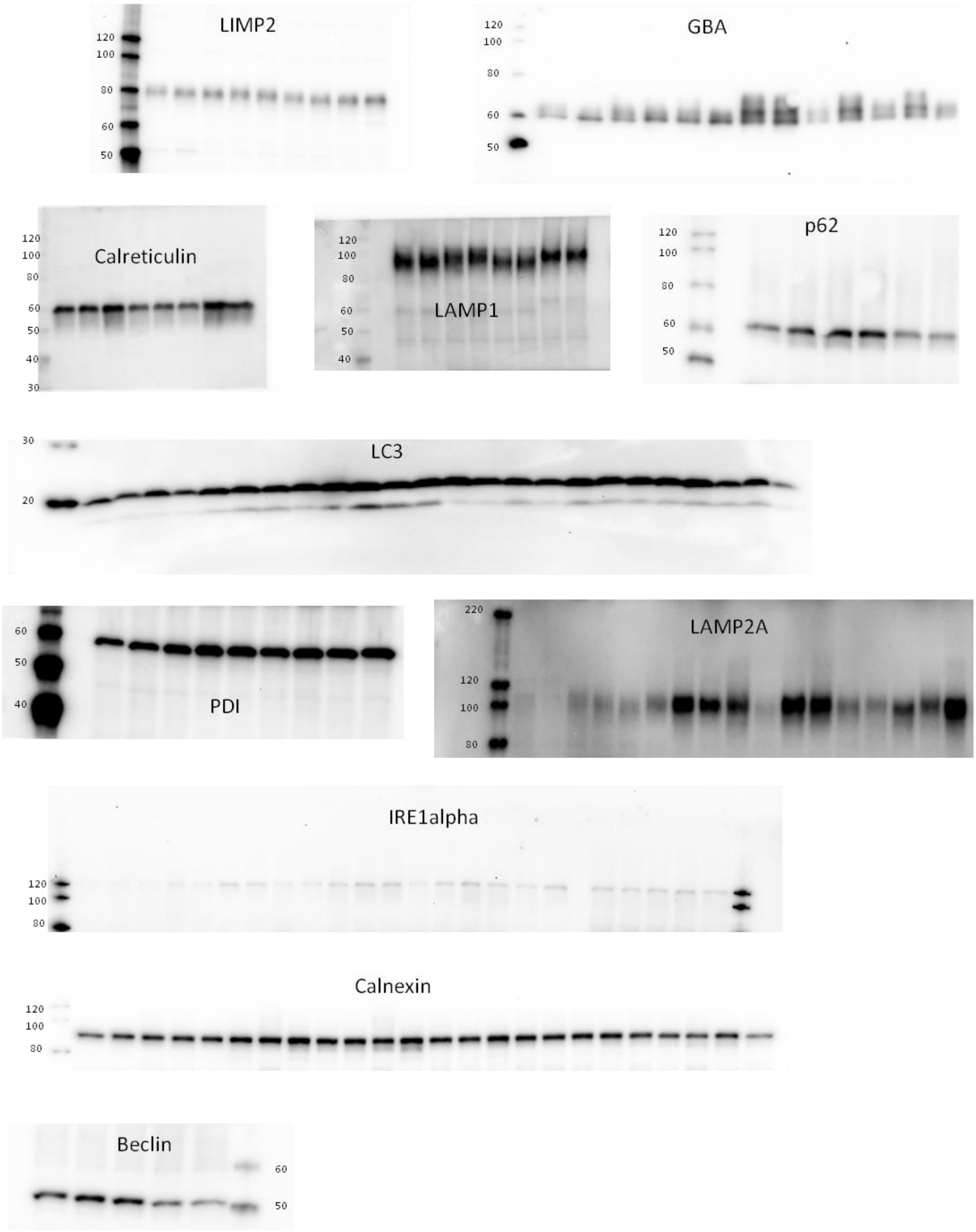

Supplemental Figures

Figure S1. Related to Figure 1. Establishment of PD iPSC lines from patient fibroblasts. (A) *GBA* N370S mutation screening. Novel XhoI restriction site identified positive cases by PCR which were then confirmed by genomic sequencing analysis – arrow indicates mutation site. (B) PD iPSC lines express expected pluripotency proteins, as shown by FACs for Tra-1-60, SSEA-4 and Nanog (grown feeder-free); open black plot represents antibody, filled grey plot represents isotype control; Right-hand panel shows the expected iPSC colony morphology, with high nucleus to cytoplasm ratio by phase-contrast microscopy; cells are densely packed when photographed on feeders, but SFC lines are photographed one day after thawing onto matrigel, so have not yet clustered tightly together. Scale bar = 100 µm. iPS-NHDF-1 (previously published) are included as a positive control reference cell line for FACs. (C) Transgene silencing in iPSC lines. qRT-PCR for each transgene, normalised to actin endogenous control, and expressed in comparison to the expression of transgenes from fibroblasts 5 days post-infection with the Yamanaka reprogramming retroviruses (HDF+Y). Uninfected fibroblasts (HDF) serve as a negative control. (D) Cytotune Sendai virus clearance in iPSC lines by RT-PCR. L, Log2 ladder; Se, Sendai backbone 181 bp; S, Sox2 451 bp; K, Klf4 410 bp; M, c-myc 532 bp; O, Oct-4 483 bp; A, β-actin control 92 bp; + control, fibroblasts infected with Cytotune 5 days previously. iPSc lines show the correct size band for β-actin, and no bands corresponding to the reprogramming virus PCR product sizes. (E) PluriTest analysis of Illumina HT12v4 transcriptome array data shows the tested PD iPSC lines cluster with the (previously published) control iPSC lines pluripotent stem cells in the red cloud and not with differentiated cells (blue clouds). Each circle represents one iPSC line.

Figure S2. Related to Figure 1. Karyotype analysis of iPSC lines used in the study. Genome integrity was assessed by Illumina Human CytoSNP-12v2.1 or OmniExpress24 SNP array and karyograms produced using KaryoStudio software (Illumina). Amplifications (green), deletions (orange) and LOH regions (grey) are shown alongside the relevant chromosome (except that in females the X chromosomes are annotated with grey, and single-copy sex chromosomes are annotated orange).

Figure S3. Related to Figure 1. Similar differentiation efficiency across lines. Neuronal and dopaminergic differentiation efficiency for control and heterozygous *GBA-N370S* mutant PD patient lines. Approximately 40% of cells developed into Tuj1-positive neurons, approximately 35% of which were Tuj1/TH double-positive. Data represent mean ± SEM of at least 3 independent differentiations. (Two-way ANOVA with Tukey post hoc analysis, $P>0.05$, not significant).

Figure S4. Related to Figures 2 and 3. (A) No accumulation of GlcCer substrate as measured by mass spectrometry quantification of individual GlcCer species normalized to the ceramide precursor. Each bar represents mean ± SEM of differentiated lines from different individuals done in triplicate (n=3). (B) No activation of XBP1 mRNA splicing events for dopaminergic neuronal cultures. After dopaminergic neuronal differentiation, mRNA was extracted and screened for XBP1 splicing events by RT-PCR for controls and heterozygous *GBA-N370S* cultures.

Figure S5. Related to Figure 6. Analysis of the lysosomal compartment. (A) No alteration for the number of lysosomes in non-TH cells in heterozygous *GBA-N370S* dopaminergic cultures when compared to controls as determined by EM analysis. (B) No differences found for the size of lysosomes in non-TH cells in heterozygous *GBA-N370S* dopaminergic cultures when compared to controls as determined by EM analysis. (C) Efficient lysosomal biogenesis in dopaminergic neurons determined by EM. 24 h starvation or 6 h bafilomycin treatment resulted in an increased number of lysosomes in TH positive neurons relatively to basal condition for both control and *GBA-N370S* dopaminergic cultures, as determined by EM quantification of LAMP1 labeled structures.

Figure S6. Related to Figure 7. Microvesicle analysis of culture media. For the microvesicle analysis of culture media no differences were observed for the size (D) or concentration (E) of exosomes between control and heterozygous GBA-N370S groups.

Figure S7. Representative western blots for antibodies used. Multiple western blots are given together with the respective associated molecular weight markers, for the clarification of the bands of the multiple proteins quantified across the manuscript.

Supplemental Table

Table S1 Summary of PD patients derived iPSC lines used in the study.

ID	Diagnosis	Genotype	Age of Biopsy (years)	Age of onset (years)	Number of iPS clones generated	ID of iPS clones used	Original IDs	Characterization
Control-1	healthy	wt/wt	44	-	2	Ctrl 1#1<br>Ctrl 1#2	NHDF-1<br>NHDF-2	Hartfield *et al* 2014
Control-2	healthy	wt/wt	36	-	3	Ctrl 2#1	OX1-19	Van Wilgenburg *et al* 2013
Control-3	healthy	wt/wt	67	-	6	Ctrl 3#1<br>Ctrl 3#2	SFC840-03-01 [AH017-11]<br>SFC840-03-03 [AH017-13]	This study
GBA-PD-1	PD	N370S/wt	81	77	4	PD 1#1<br>PD 1#2	MK071-1<br>MK071-3	This study
GBA-PD-2	PD	N370S/wt	46	40	3	PD 2#1	MK088-1	This study
GBA-PD-3	PD	N370S/wt	72	69	3	PD 3#1<br>PD 3#2	SFC834-03-01<br>SFC834-03-03	This study

Supplemental Experimental Procedures:

Participant recruitment

Participants were recruited to this study having given signed informed consent, which included mutation screening and derivation of hiPSC lines from skin biopsies (Ethics committee: National Health Service, Health Research Authority, NRES Committee South Central – Berkshire, UK, who specifically approved this part of the study - REC 10/H0505/71). All the patients included in our study fulfilled UK Brain Bank diagnostic criteria for clinically probable PD at presentation (Hughes et al., 1992). *GBA*-associated parkinsonism is known to present and subsequently progress in a different way from patients with sporadic PD. In general, a younger age of onset, increased risk of early Parkinson's-related dementia, early falls and postural imbalance and initial dementia phenotype are recognized with heterozygous GBA mutations (Asselta et al., 2014; Gan-Or et al., 2015; Winder-Rhodes et al., 2013). Patients 1 and 2 presented with akinetic-rigid parkinsonism, and maintained a good levodopa-response for their first 5 years of treatment without significant falls or dementia. Patient 3 presented with akinetic-rigid parkinsonism, failed to respond to dopaminergic medication and subsequently rapidly progressed more quickly with early dementia and frequent falls two years later.

***GBA*-N370S mutation screening**

Study participants were screened for the N370S mutation in the GBA gene by extracting genomic DNA from blood samples using the AutoPure LS® Kit (QIAGEN). PCR reactions were carried out using AmpliTaq Gold DNA polymerase (Applied Biosystems). Primer sequences were used as follows: 5'-GCCTTTGTCCTTACCCTC*G -3' and 5'- GACAAAGTTACGCACCCAA-3'. * indicates a mismatch that was engineered into the forward primer in order to create a XhoI restriction site in the PCR product from participants carrying the N370S mutation as described previously(Aharon-Peretz et al., 2004). The resulting PCR product was digested with XhoI (NEB) and resolved by agarose gel electrophoresis. Mutations were confirmed by sequencing. Briefly, DNA was treated with an ExoSAP reaction as follows: 1X SAP buffer, shrimp alkaline phosphatase (500 U; SAP, Promega), Exonuclease I (2 U; NEB). Samples were incubated at 37 °C for 1 hour and then at 80 °C for 20 minutes. The sequencing reaction was performed according to BigDye Terminator v3.1 Cycle Sequencing protocol (Applied Biosystems). Following a clean up step, the sequencing read was performed on a 3700 DNA Analyser (Applied Biosystems) sequencing platform.

Culture and reprogramming of primary fibroblasts

All iPSC lines used in this study were derived from skin biopsies and reprogrammed using the same methodology and in the same laboratory. Skin punch biopsies (4 mm in diameter) were obtained from participants and the subcutaneous tissue removed. The dermal sample was cut into several small pieces and cultured in ADMEM (Invitrogen) supplemented with USDA-approved foetal bovine serum (10%; Sigma) and penicillin/streptomycin (1%) in a humidified incubator (37 °C, 5% CO_2). Glass coverslips were placed over the biopsy to facilitate adhesion. Fibroblast were reprogrammed at passage 3-5, using either a) reprogramming retroviruses carrying individual reprogramming genes (pMXs plasmids encoding the reprogramming genes were obtained from Addgene -17220: pMXs-hc-MYC, 17219: pMXs-hKLF4, 17218: pMXs-hSOX2, 17217: pMXs-hOCT3/4, 13354: pMXs-Nanog) as in the original papers by Takahashi and Yamanaka(Kitamura et al., 2003; Takahashi et al., 2007; Takahashi and Yamanaka, 2006). They were packaged using the Plat-GP retroviral packaging cell line (containing retroviral gag and pol genes) and cotransfected with VSV-G, using PEI, to make iPS retrovirus vectors. Reprogramming was carried out following[4]; Specifically, 50,000 fibroblasts were infected on days 0 and 1 with an equal volume of each virus supernatant except Oct which was used at a 3-fold dose, plus 5 µg/ml polybrene and spinoculation (1200 g for 45 minutes at 16°C). Or b) One control donor (SFC840) and one GBA donor (SFC834) was reprogrammed using Cytotune Sendai virus reprogramming kit (Invitrogen) according to the manufacturer's instructions, scaled down to infect 50,000 fibroblasts at the same recommended MOI. For both reprogramming methods, fibroblasts were transferred onto mitotically-inactivated 'Pathology Oxford' (Gardner, 1982) outbred mouse embryonic feeder cells (MEF) on 0.1% gelatin coated plates (Sigma) on day 4, and from day 5 onwards were cultured in standard KnockOut serum replacement medium (Knock-out DMEM (Invitrogen), KO-Serum Replacement (20% Invitrogen), Glutamax-I (2 mM Invitrogen), non-essential amino acids (1%, Invitrogen), penicillin (100 U/mL Invitrogen), streptomycin (100 µg/mL Invitrogen), 2-ME (55 µM Invitrogen) and bFGF (10 ng/mL R&D)) (supplemented with 50 µg/ml ascorbic acid and 0.5 µM Valproic acid (both from Sigma) for the retroviral protocol to enhance reprogramming efficiency),

replacing 50% medium on alternate days, and substituting with MEF-conditioned medium from day 10 onwards. Colonies displaying iPSC morphology were picked on day ~28 and passaged on MEFs by manual dissection every 5-7 days. iPSC lines were adapted to feeder-free culture conditions by three passages on Matrigel coated plates (BD Matrigel hESC-qualified Matrix) in mTeSR™1 (StemCell Technologies), using 0.5 mM EDTA in PBS to dissociate cells to small clumps (Beers et al., 2012). For some applications, enzymatic dissociation (TryplE Express, Gibco) supplemented with Rock inhibitor Y27632 (10 µM; Calbiochem) on the day of passage was used. – in this case, the number of feeder-free passages was always kept to an absolute minimum to reduce the likelihood of genetic change. Cells were frozen in SNP-QCed batches of at least 30 vials (within a narrow window of passage numbers, ~p15-30), from which cells would be thawed for each experiment, to ensure consistency across experiments.

Characterisation of iPSCs

Morphological assessment, passageability and immunocytochemistry for markers of pluripotency were carried out as initial indicators of pluripotency. After converting hiPSCs to feeder-free culture on Matrigel (BD Biosciences) for at least 3 passages, cells were harvested for more in depth characterisation analyses and RNA and genomic DNA was made using an All-Prep kit (Qiagen).

For fluorescence activated cell sorting (FACs) of pluripotency markers the following antibodies were used, with appropriate isotype control, at the same concentration, from the same supplier (clone, isotype control, supplier): TRA-1-60 (B119983, IgM-488, Biolegend), SSEA-4-633 (MAB1435, IgG3-488, R&D), on fresh cells, or, for staining in combination with Nanog (2985S, IgG-647, Cell Signaling), they were fixed for 10 minutes in 2% paraformaldehyde in PBS (Alfa Aesar), permeabilised in 100% methanol at -20 for at least 30 mins before staining,. Fluorescence was measured using a FACS Calibur (Becton Dickinson), and data was analysed using FlowJo software.

qRT-PCR for assessing the degree of silencing of retroviral transgene sequences was carried out using primer sequences published by Takahashi et al. supplementary table S12[4], except that in place of the published reverse primer (pMXs-AS3200 TTA TCG TCG ACC ACT GTG CTG CTG), we used our own designed primer, pMXs-AS3200v2 (TTA TCG TCG ACC ACT GTG CTG GCG) which had exactly the same sequence as the target pMXs vector backbone sequence and which therefore amplified more efficiently; also, for amplifying transgene mNanog, the forward primer GCT CCA TAA CTT CGG GGA GG. RNA was reverse transcribed using a RetroScript kit (Ambion), using 2ug template RNA in 20 µl reaction volume. 2 µl of 1:10 dilution of cDNA product was used in a 25 µl qRT-PCR reaction. QRT-PCR was carried out on an Applied Biosystems StepOne Plus Real Time PCR machine, with StepOne software, using Applied Biosystems 2xSYBR green PCR mix + ROX and 60°C anneal, Target gene transcript levels were compared to actin B control (actin B primers, Eurogentec), and subsequently to fibroblasts harvested 5 days after infection with the reprogramming vectors.

RT-PCR to assess clearance of Cytotune Sendai virus-delivered reprogramming genes was preformed according to the manufacturer's instructions and run on a 1.5% agarose gel with Log2 ladder (NEB). Positive controls (fibroblasts infected 5 days previously) were always run in parallel. Primers were SeV F: GGATCACTAGGTGATATCGAGC, R: ACCAGACAAGAGTTTAAGAGATATGTATC 181bp; SOX2 F: ATGCACCGCTACGACGTGAGCGC, R: AATGTATCGAAGGTGCTCAA 451bp; KLF4 F: TTCCTGCATGCCAGAGGAGCCC, R: AATGTATCGAAGGTGCTCAA 410bp; c-MYC F: TAACTGACTAGCAGGCTTGTCG, R: TCCACATACAGTCCTGGATGATGATG 532bp; OCT4 F: CCCGAAAGAGAAAGCGAACCAG, R: AATGTATCGAAGGTGCTCAA 483bp; β-Actin control Eurogentec 92bp

Genome integrity was assessed by an Illumina Human CytoSNP-12v2.1 beadchip array (~300,000 markers) or OmniExpress24 array (700,000 markers, SFC840 and SFC834 lines) and analyzed using KaryoStudio and GenomeStudio software (Illumina), comparing the iPSC lines to the parental fibroblasts. Note that one of the parental fibroblast lines was a mosaic (a proportion of GBA-PD-2 cells having trisomy 8), but the derived iPSc line used in this study was a normal karyotype. Clinical records indicated that there was no known close relatedness between the individuals used in this study.

Analysis of pluripotency was performed on RNA extracted from iPSC lines using the Illumina HT12v4 transcriptome array. The image data files were then uploaded to www.pluritest.org and scored for pluripotency, as previously described (Müller et al., 2011). According to this analysis, pluripotent cell lines will cluster in the top left quadrant of the graph, indicating high pluripotency score and low novelty score (novelty meaning genes

that are not commonly expressed in iPSCs). All lines tested had acceptable pluripotency scores, but note that one line (iPS-MK088-1) appeared by this assessment to express more ‘novelty genes’ than the other lines – however, this was not reflected in its passageability, or the other assays undertaken, nor in the ability of the lines to differentiate to Embryoid Bodies (EBs) and neuronal lineages.

Differentiation of DA neurons

Prior to differentiation, iPSC lines were adapted to feeder-free conditions using Matrigel (BD). mTeSR™1 (StemCell Technologies) was supplemented with Y27632 (10μM; Calbiochem) on the day of passage. Embryoid bodies (EBs) were formed by dissociation of iPSCs with TryplE and seeded into Aggrewell plates (10,000 cells per EB; Stem Cell Technologies) in mTeSR™-1 medium supplemented with Y27632 (10 µM), with a 75% daily medium change. After 4 days, neural induction was initiated.

All materials for differentiation were obtained from Life Technologies unless otherwise stated. EBs were plated onto Geltrex-coated plates in Neural Induction medium 1 (DMEM/F12 supplemented with L-glutamine [2 mM], N2 supplement, bovine serum albumin [1 mg/ml], Y27632 [10 µM; Tocris], SB431542 [10 µM, Tocris], noggin [200 ng/ml], sonic hedgehog [500 ng/ml; SHH C24II; R&D Systems], CHIR99021 [0.7 µM; Stemgent] and antibiotic/ antimycotic [1% v/v]). After 12 days, medium was changed to Neural Induction medium 2 (as NI1, without SB431542 and noggin, SHH C24II [20 ng/ml], FGF8a [100 ng/ml; R&D Systems], heparin [5 µg/ml; Sigma], BDNF [20 ng/ml] and ascorbic acid [200 µM; Sigma]) and incubated for 8 days, until the appearance of dense neural rosette structures became apparent. Neural progenitor cells were manually selected and replated onto poly-D-lysine/laminin-coated plates in final differentiation medium (DMEM/F12 supplemented with L-glutamine [2 mM], N2 supplement, BDNF [20 µg/ml], glial-derived neurotrophic factor [GDNF, 20 µg/ml], N^6,2′-O-dibutyryladenosine 3′,5′-cyclic monophosphate sodium salt [dCAMP, 0.5 mM; Sigma], laminin [1 µg/ml] and antibiotic/antimycotic (1% [v/v]). Neurons were matured for 2-6 weeks in this medium before experimental procedures were carried out. For additional experiments (Figures 2E, 7E and S4), cells were differentiated as previously described (Kriks et al., 2011)

Immunocytochemistry

Cells were fixed in 4% paraformaldehyde and permeabilised in 0.1% Triton-X100 prior to immunostaining. Coverslips were blocked in 10% goat or donkey serum for 1 hour before incubating with primary antibodies overnight at 4°C. Antibodies used as follows: FoxA2 (1:500; R&D systems), TUJ1 (β-3 tubulin) (1:500; Covance), TH (1:500; Millipore), LC3 (1:200; Nanotools), PiTX3 (1:100, Life Technologies) and Nurr1 (1:400, Millipore). Secondary antibodies (Alexa fluor, Life Technologies) were incubated for 1 hour at room temperature before mounting and analysis. Images were captured using a Leica SP5 confocal or a EVOS FL microscope.

RT-PCR analysis

RNA was extracted from cells using Trizol (Life Technologies) and purified using the RNeasy kit (QIAGEN). Reverse transcription was performed using Superscript III (Life Technologies) according to manufacturer’s instructions. Polymerase chain reactions (PCR) were set up with 20 ng of cDNA using GoTaq DNA Polymerase products (Promega). Primer sequences used as follows: FOXA2: 5’-GACAAGTGAGAGAGCAAGTG-3’ and 5’-ACAGTAGTGGAAACCGGAG-3’; LMX1A: 5’-AACGACAGCTTCTGGCATGA-3’ and 5’-TCAAGATGGTTCTCGGACGT-3’; EN1: 5’-GCTATCCTACTTATGGGCTCA-3’ and 5’-GGAGTGGTTGTACAGTCCCT-3’; NURR1: 5’-CGACATTTCTGCCTTCTCC-3’ and 5’-GGTAAAGTGTCCAGGAAAAG-3’; OCT4: 5’-AAAGCTCTGCAGAAAGAACTCG-3’ and 5’-CTCACTCGGTTCTCGATACTGG-3’; GAPDH: 5’-CAGGGCTGCTTTTAACTCTGG-3’ and 5’-AAGTTGTCATGGATGACCTTGG-3’.

Western blot

Western blotting was carried out on whole cell lysates extracted using RIPA buffer (Tris [50 mM, pH 8], sodium chloride [150 mM], sodium dodecyl sulphate [SDS; 0.1% w/v], sodium deoxycholate [0.5 % w/v] and nonidet-P40 [1% w/v]). Before loading, samples were denatured for 5 minutes at 100 °C. Protein separation was achieved using SDS polyacrylamide gel electrophoresis and transferred onto PVDF membrane. Antibodies used as follows: TH (1:500; Millipore, AB1542), GBA (1:200; Abcam, ab55080), β-actin (1:10,000; Abcam, ab8227), LIMP2 (1:1000; ProSci, 4621), BiP (1:200; Abcam, ab21685), LC3 (1:200; Nanotools, 5F10), Beclin-1 (1:500; Abcam, ab51031), α-synuclein (1:200; Covance, SIG-39730-200), Cathepsin D (1:500; Abcam, ab6313), P62 (1:500; Abcam, ab56416).

Immunogold electron microscopy

Cells grown on polyester filters were fixed in 3% paraformaldehyde/0.05% glutaraldehyde and prepared for immunogold EM by standard methods (Morris et al., 2006). Filters were stained with uranyl acetate (2 % w/v in distilled water), dehydrated through increasing concentrations of methanol (70-100 %) and embedded in LR Gold resin (Agar, Reading UK). Ultra-thin sections (50-80 nm) were prepared by use of a Reichert Ultracut S ultratome (Leica, Milton Keynes, UK), mounted on 200-mesh nickel grids, incubated at room temperature with either anti-profilin antibody (dilution 1:200, 2 hour) or anti-TH antibody (1:200, 2 hour) followed by Protein A-15 nm gold complex for 1 hour (1:60). All antisera were diluted in 0.1M phosphate buffer containing 0.1 % egg albumin. As a negative control, the primary antibody was replaced by non-immune sera and immunogold labelling was not observed. After immunolabelling sections were lightly counterstained with lead citrate and uranyl acetate and examined with a JEOL transmission electron microscope (JEM-1010, JEOL, Peabody, MA, USA) and representative micrographs were prepared. The area of the cells was analysed using Axiovison (version 4.5) image analysis software and the number of lysosomes counted for 8 cells per group**.** Lysosomes were manually counted by an investigator blinded to genotype and treatment. All blocks were coded, labeled, and quantified prior to counting and analysis. Decoding was only done when all analysis was complete.

GCase activity assay

GCase activity was measured as described previously. Briefly neurons were lysed and sonicated in GCase lysis buffer (citrate phosphate buffer supplemented with 0.25 % (v/v) Triton-X100 and 0.25% (w/v) taurocholic acid pH 5.4). Following incubation on ice for 30 minutes, samples were centrifuged for 10 minutes at 4°C. The supernatant was incubated with 4-methylumbelliferyl β-D-glucopyranosidase (5 mM) and incubated at 37°C for 1 hour. The reaction was stopped by adding excess glycine phosphate buffer (0.2 M, pH 10.4) and resulting fluorescence detected on a Synergy HT plate reader (BioTek). Excitation at 360 nm and emission 440 nm was used. Samples treated with conduritol B epoxide (2.5 mM; Enzo Life Sciences) were used as a negative control and to provide background values for GCase activity.

Exosome isolation

Conditioned media from iPSC-derived dopaminergic neuronal cultures were subjected to serial centrifugation at 1,500 g for 10 min followed by 17,000 g for 30 min with all steps performed at 4 °C. The resulting supernatant was filtered using a 0.2 μm filter and spun at 160,000 g for 1 h using a MLS-50 swinging rotor. The pellet from each group was resuspended in Hanks Balanced Salt Solution and aliquoted for Nanoparticle Tracking Analysis, and immunoblotting. For immunoblotting, 2.2x109 microvesicles were loaded per condition, which would correspond to microvesicles extracted from about 12 ml of conditioned medium from each patient's iPSC line.

Nanoparticle Tracking Analysis

Nanoparticle Tracking Analysis (NTA) uses light scattering to enable direct, real-time visualisation and analysis of nanoparticles and uses the rate of Brownian motion to estimate individual particle size. NTA is a useful technique for making rapid size and concentration measurements of microvesicle/exosome preparations because requirements of sample preparation are simple. This is an established method to measure size and concentration in the exosome field (Gardiner et al., 2013). Exosome size and concentration were assessed using a NS500

instrument (Nanosight Ltd. Amesbury, UK) equipped with a 405 nm laser and a CMOS camera. Samples were diluted in filtered PBS immediately prior to use. 5 x 30 second videos were recorded for each sample (camera gain 350; shutter speed 14.99 ms). Videos were analysed using NTA software (version 2.3). Instrument calibration was verified by analyzing silica microspheres (Polysciences, Warrington, PA) prior to each analysis.

α -syn ELISA

Briefly, each ELISA plate (Corning Costar) was coated for 24 hrs at room temperature with 0.5 μg/ml of Syn-1 antibody (50 μl per well) in 100 mM NaHCO3, pH 9.3. The plates were washed three times in wash buffer (50 mM Tris-HCl, 150 mM NaCl and 0.04% Tween-20) and 50 μl of sample was added. Recombinant human α-Syn (Chemicon) (as standard) was diluted in 50% TBST/BSA (10 mM Tris-Cl, pH 7.6, 100 mM NaCl, 0.1% Tween-20 and 1% BSA) and 50% cell culture medium. Samples of conditioned medium were 2-fold diluted in TBST/BSA prior to addition to the wells. To allow antigen binding, plates were incubated at 37 ^{o}C for 2.5 hrs. After washing three times with wash buffer, 50 μl of HRP-conjugated C-20 antibody (1:4000 diluted in TBST/BSA) was added to each well and further incubated for 1 hr at room temperature. The wells were washed and 50 μl of chemiluminogenic HRP substrate (UptiLight HS ELISA HRP substrate, Interchim) was added to each well. Following incubation for 10 minutes at room temperature, chemiluminescence was integrated for 1s. Standards and conditioned medium samples were measured at least in duplicate.

α -syn Meso Scale Discovery (MSD)

Conditioned media samples (100 μl) from iPSC-derived dopaminergic neuronal cultures were loaded on commercial available human α–synuclein plates purchased from Meso Scale Discovery (Rockville, MD, USA, Cat# K151TGD-2). Assay was performed following the manufacturer's instructions and read using a MESO QuickPlex SQ 120 instrument (Meso Scale Discovery).

Glucosylceramide (GlcCer) and ceramide (Cer) quantification.

Quantitative analysis of sphingolipids was performed using liquid chromatography and tandem mass spectrometry as described previously (Gegg et al., 2015). Briefly, cell pellets were extracted with 0.5 mL of a solution of acetonitrile:methanol:water (97:2:1, v/v/v). Samples were analyzed with an AB Sciex API-5000 mass spectrometer (AB Sciex, Framingham, MA).

Statistics

Results are expressed as mean ± SEM from performing at least three independent differentiation experiments per line each analysed in triplicate except otherwise stated. Statistical significance was determined by Student's *t*-test or with two-way ANOVA.

Supplemental References:

Aharon-Peretz, J., Rosenbaum, H., and Gershoni-Baruch, R. (2004). Mutations in the glucocerebrosidase gene and Parkinson's disease in Ashkenazi Jews. The New England journal of medicine *351*, 1972-1977.

Asselta, R., Rimoldi, V., Siri, C., Cilia, R., Guella, I., Tesei, S., Solda, G., Pezzoli, G., Duga, S., and Goldwurm, S. (2014). Glucocerebrosidase mutations in primary parkinsonism. Parkinsonism & related disorders *20*, 1215-1220.

Beers, J., Gulbranson, D., George, N., Siniscalchi, L., Jones, J., Thomson, J., and Chen, G. (2012). Passaging and colony expansion of human pluripotent stem cells by enzyme-free dissociation in chemically defined culture conditions. Nat Protocols *7*, 2029-2040.

Gan-Or, Z., Amshalom, I., Kilarski, L.L., Bar-Shira, A., Gana-Weisz, M., Mirelman, A., Marder, K., Bressman, S., Giladi, N., and Orr-Urtreger, A. (2015). Differential effects of severe vs mild GBA mutations on Parkinson disease. Neurology *84*, 880-887.

Gardiner, C., Ferreira, Y.J., Dragovic, R.A., Redman, C.W., and Sargent, I.L. (2013). Extracellular vesicle sizing and enumeration by nanoparticle tracking analysis. Journal of extracellular vesicles *2*.

Gardner, R.L. (1982). Investigation of cell lineage and differentiation in the extraembryonic endoderm of the mouse embryo. Journal of embryology and experimental morphology *68*, 175-198.

Gegg, M.E., Sweet, L., Wang, B.H., Shihabuddin, L.S., Sardi, S.P., and Schapira, A.H. (2015). No evidence for substrate accumulation in Parkinson brains with GBA mutations. Movement disorders : official journal of the Movement Disorder Society *30*, 1085-1089.

Hughes, A.J., Daniel, S.E., Kilford, L., and Lees, A.J. (1992). Accuracy of clinical diagnosis of idiopathic Parkinson's disease: a clinico-pathological study of 100 cases. Journal of neurology, neurosurgery, and psychiatry *55*, 181-184.

Kitamura, T., Koshino, Y., Shibata, F., Oki, T., Nakajima, H., Nosaka, T., and Kumagai, H. (2003). Retrovirus-mediated gene transfer and expression cloning: powerful tools in functional genomics. Experimental hematology *31*, 1007-1014.

Kriks, S., Shim, J.W., Piao, J., Ganat, Y.M., Wakeman, D.R., Xie, Z., Carrillo-Reid, L., Auyeung, G., Antonacci, C., Buch, A., *et al.* (2011). Dopamine neurons derived from human ES cells efficiently engraft in animal models of Parkinson's disease. Nature *480*, 547-551.

Morris, J.F., Omer, S., Davies, E., Wang, E., John, C., Afzal, T., Wain, S., Buckingham, J.C., Flower, R.J., and Christian, H.C. (2006). Lack of annexin 1 results in an increase in corticotroph number in male but not female mice. Journal of neuroendocrinology *18*, 835-846.

Müller, F.-J., Schuldt, B.M., Williams, R., Mason, D., Altun, G., Papapetrou, E.P., Danner, S., Goldmann, J.E., Herbst, A., Schmidt, N.O., *et al.* (2011). A bioinformatic assay for pluripotency in human cells. Nature methods *8*, 315-317.

Takahashi, K., Tanabe, K., Ohnuki, M., Narita, M., Ichisaka, T., Tomoda, K., and Yamanaka, S. (2007). Induction of Pluripotent Stem Cells from Adult Human Fibroblasts by Defined Factors. Cell *131*, 861-872.

Takahashi, K., and Yamanaka, S. (2006). Induction of pluripotent stem cells from mouse embryonic and adult fibroblast cultures by defined factors. Cell *126*, 663-676.

Winder-Rhodes, S.E., Evans, J.R., Ban, M., Mason, S.L., Williams-Gray, C.H., Foltynie, T., Duran, R., Mencacci, N.E., Sawcer, S.J., and Barker, R.A. (2013). Glucocerebrosidase

mutations influence the natural history of Parkinson's disease in a community-based incident cohort. Brain : a journal of neurology *136*, 392-399.
