# Supplementary material for: ER Stress and Autophagic Perturbations Lead to Elevated Extracellular α-Synuclein in GBA-N370S Parkinson's iPSC-Derived Dopamine Neurons
Source: Stem Cell Reports. 2016 Feb 18;6(3):342–56. doi: 10.1016/j.stemcr.2016.01.013 (PMC4788783; doi:10.1016/j.stemcr.2016.01.013)
Supplement: Document S2. Article plus Supplemental Information [file mmc2.pdf]

Stem Cell Reports
Article
OPEN ACCESS

ER Stress and Autophagic Perturbations Lead to Elevated Extracellular α-Synuclein in *GBA-N370S* Parkinson's iPSC-Derived Dopamine Neurons

Hugo J.R. Fernandes,[1,2] Elizabeth M. Hartfield,[1,2] Helen C. Christian,[2] Evangelia Emmanoulidou,[3] Ying Zheng,[1,4] Heather Booth,[1,2] Helle Bogetofte,[1,2,5] Charmaine Lang,[1,2] Brent J. Ryan,[1,2] S. Pablo Sardi,[6] Jennifer Badger,[1,2] Jane Vowles,[1,7] Samuel Evetts,[1,4] George K. Tofaris,[1,4] Kostas Vekrellis,[3] Kevin Talbot,[1,4] Michele T. Hu,[1,4] William James,[1,7] Sally A. Cowley,[1,7] and Richard Wade-Martins[1,2,*]
[1]Oxford Parkinson's Disease Centre
[2]Department of Physiology, Anatomy and Genetics
University of Oxford, South Parks Road, Oxford OX1 3QX, UK
[3]Division of Basic Neurosciences, Biomedical Research Foundation of the Academy of Athens, Athens 11526, Greece
[4]Nuffield Department of Clinical Medicine, Division of Clinical Neurology, University of Oxford, Oxford OX3 9DU, UK
[5]Institute of Molecular Medicine, University of Southern Denmark, Odense 5230, Denmark
[6]Genzyme, a Sanofi Company, Framingham, MA 01701, USA
[7]The James Martin Stem Cell Facility, Sir William Dunn School of Pathology, University of Oxford, South Parks Road, Oxford OX1 3RE, UK
*Correspondence: richard.wade-martins@dpag.ox.ac.uk
http://dx.doi.org/10.1016/j.stemcr.2016.01.013
This is an open access article under the CC BY license (http://creativecommons.org/licenses/by/4.0/).

SUMMARY

Heterozygous mutations in the glucocerebrosidase gene (*GBA*) represent the strongest common genetic risk factor for Parkinson's disease (PD), the second most common neurodegenerative disorder. However, the molecular mechanisms underlying this association are still poorly understood. Here, we have analyzed ten independent induced pluripotent stem cell (iPSC) lines from three controls and three unrelated PD patients heterozygous for the *GBA-N370S* mutation, and identified relevant disease mechanisms. After differentiation into dopaminergic neurons, we observed misprocessing of mutant glucocerebrosidase protein in the ER, associated with activation of ER stress and abnormal cellular lipid profiles. Furthermore, we observed autophagic perturbations and an enlargement of the lysosomal compartment specifically in dopamine neurons. Finally, we found increased extracellular α-synuclein in patient-derived neuronal culture medium, which was not associated with exosomes. Overall, ER stress, autophagic/lysosomal perturbations, and elevated extracellular α-synuclein likely represent critical early cellular phenotypes of PD, which might offer multiple therapeutic targets.

INTRODUCTION

Parkinson's disease (PD) is the second most common neurodegenerative disorder, characterized by the preferential degeneration of dopamine neurons in the substantia nigra pars compacta (SNpc). Heterozygous mutations in the glucocerebrosidase gene (*GBA*) encoding the lysosomal enzyme GCase represent the strongest common genetic risk factor for PD (Sidransky et al., 2009) and have also been associated with other related Lewy body disorders (Goker-Alpan et al., 2006); however, the underlying molecular mechanisms are still poorly understood.

The association of *GBA* with PD first emerged from clinical studies that demonstrated that relatives of patients with Gaucher's disease (GD), a lysosomal storage disease caused by homozygous *GBA* mutations, had an increased incidence of PD (Goker-Alpan et al., 2004). More recent studies exploring the pathogenic role of homozygous *GBA* mutations in GD have highlighted a role of GCase in mitochondria function, α-synuclein aggregation, and autophagic machinery (Mazzulli et al., 2011; Sardi et al., 2011). GCase pathology in the context of heterozygous *GBA* mutations in PD have been addressed in recent postmortem (Gegg et al., 2012) and patient fibroblast (McNeill et al., 2014) studies, and recently in a PD patient human neuronal model (Schöndorf et al., 2014). Overall, mutations in *GBA* and in *LRRK2*, also known to play a role in regulating autophagy (Alegre-Abarrategui et al., 2009), have emphasized the role for the autophagic/lysosomal pathway as central to the pathogenesis of PD (Tofaris, 2012).

Human induced pluripotent stem cells (iPSCs) derived from patients carrying disease-associated alleles retain the genetic background likely to include disease-permissive genetic modifiers and can be differentiated into highly physiological models of specific cell types to study cellular mechanisms of disease (Kiskinis and Eggan, 2010). Within this paradigm, iPSCs can be differentiated into functional midbrain dopamine neurons to provide a powerful tool to study the genetic contribution to PD (Hartfield et al., 2012). We have previously developed a highly physiological cellular model of differentiated midbrain dopamine neurons that express key dopaminergic markers, exhibit dopamine synthesis, release, and re-uptake, and show autonomous pace-making and spontaneous synaptic activity (Hartfield et al., 2014).

Here we study the impact of the common *GBA-N370S* mutation on the phenotype of patient-specific dopaminergic neuronal cultures differentiated from iPSC lines

derived from patients with PD and identify deficits in protein homeostasis. Our results suggest that the heterozygous *GBA-N370S* mutation leads to a cellular gain of function, shown by misprocessing of misfolded GCase protein in the ER resulting in ER stress upregulation and autophagic/lysosomal dysfunction leading to an enlargement of the lysosomal compartment in individually identified vulnerable dopamine neurons. Together, these deficits lead to increased release of extracellular α-synuclein from GBA-*N370S* PD human iPSC-derived dopamine neuron cultures, which we show is not associated with exosomes. We propose that the combination of these disturbances impairs protein homeostasis in dopamine neurons contributing to their preferential vulnerability in PD. These data highlight the early pathogenic relevance of GCase function in the autophagic/lysosomal pathway in PD, and may explain the higher risk for heterozygous *GBA-N370S* mutation carriers to develop PD.

RESULTS

Generation of Human iPSCs

PD patients and controls from the Discovery clinical cohort established by the Oxford Parkinson's Disease Centre (OPDC) were screened for the presence of *GBA-N370S* heterozygous mutation (Figure S1A). We then generated and characterized 22 human iPSC clonal lines from fibroblasts obtained from three unrelated PD patients carrying a *GBA-N370S* heterozygous mutation and three healthy control individuals (Table S1). Detailed characterization of all PD lines used in this study is shown in Figures S1 and S2. Characterization of lines from two of the control individuals has been published previously (Control-2, van Wilgenburg et al., 2013; Control-1, Hartfield et al., 2014). All iPSC lines displayed embryonic stem cell-like morphology and expressed pluripotency-associated proteins (TRA-1-60, SSEA-4, and Nanog; Figure S1B). Silencing of retroviral transgenes upon establishment of pluripotency was confirmed by qRT-PCR (Figure S1C), and Sendai virus-reprogrammed lines were checked for clearance of the exogenous genes by RT-PCR (Figure S1D). Pluripotency was also assessed using the PluriTest, which is based upon analysis of transcriptome data from iPSCs and human embryonic stem cells and compares gene expression with a large reference set of genome-wide profiles from multiple cell and tissue types (Müller et al., 2011). Accordingly, all lines used were classified as fully reprogrammed and pluripotent (Figure S1E). Genome integrity was confirmed by Illumina SNP arrays (Figure S2) providing detailed resolution of genome integrity compared with traditional karyotyping or M-FISH. The SNP datasets also enabled confirmation that the iPSC lines derived from the expected fibroblast line. The SNP datasets and the Illumina HT12v4 transcriptome array results have been deposited in GEO: GSE53426.

Characterization of Dopaminergic Neuronal Cultures

In order to study the effect of the heterozygous *GBA-N370S* mutation in the context of PD, iPSCs were differentiated into dopaminergic neuronal cultures (Figure 1A). A total of ten different iPSC clonal lines from three control individuals and three PD patients carrying a *GBA-N370S* heterozygous mutation were differentiated into dopaminergic neuronal cultures, as described previously (Hartfield et al., 2014), with minor modifications. Briefly, embryoid bodies prepared from iPSCs were plated in the presence of SMAD signaling inhibitors (Noggin and SB431542) to initiate neuronal induction, together with CHIR99021 (a potent canonical WNT signaling activator), sonic hedgehog (SHH), and FGF8a for midbrain floor plate induction. By day 20, visible neural rosette structures were manually dissected and re-plated for differentiation into dopaminergic neurons with ascorbic acid, cAMP, brain-derived neurotrophic factor, and glial cell line-derived neurotrophic factor for 15 days. All lines successfully differentiated into dopaminergic neuronal cultures (Figure 1B). The differentiation efficiency, assessed by β-3 tubulin and TH expression using immunofluorescence, was similar across the genotypes used in the study (Figure S3). RT-PCR analysis demonstrated that differentiated dopaminergic cultures expressed the floor plate markers FOXA2 and LMX1A, in addition to the mature dopaminergic neuron markers EN1 and NURR1, while the level of the pluripotency marker OCT4 was much reduced on differentiation (Figure 1C). We confirmed the co-expression of TH with the floorplate marker FOXA2, the marker of the A9 dopaminergic neurons susceptible in PD GIRK2, and the post-mitotic midbrain neuronal marker PITX3, further confirming the mature midbrain identity of the differentiated dopaminergic neurons (Figure 1C). We have previously confirmed the highly mature, functional dopaminergic phenotype in neurons derived from iPSCs using this protocol (Hartfield et al., 2014). Neurons exhibit the correct pharmacological and physiological characteristics expected of mature dopamine neurons, including expression of a wide range of midbrain markers, dopamine synthesis and uptake, spontaneous slow autonomous Ca^{2+} pace-making activity, and spontaneous synaptic activity at 5–10 Hz, typical of dopamine neurons of the substantia nigra pars compacta (Hartfield et al., 2014).

GBA-N370S Leads to Aberrant GCase Post-translation Modification and Abnormal Lipid Profiles

Once we had established a human dopaminergic neuronal culture model consistent between different lines from multiple individuals, we next determined the effect of the

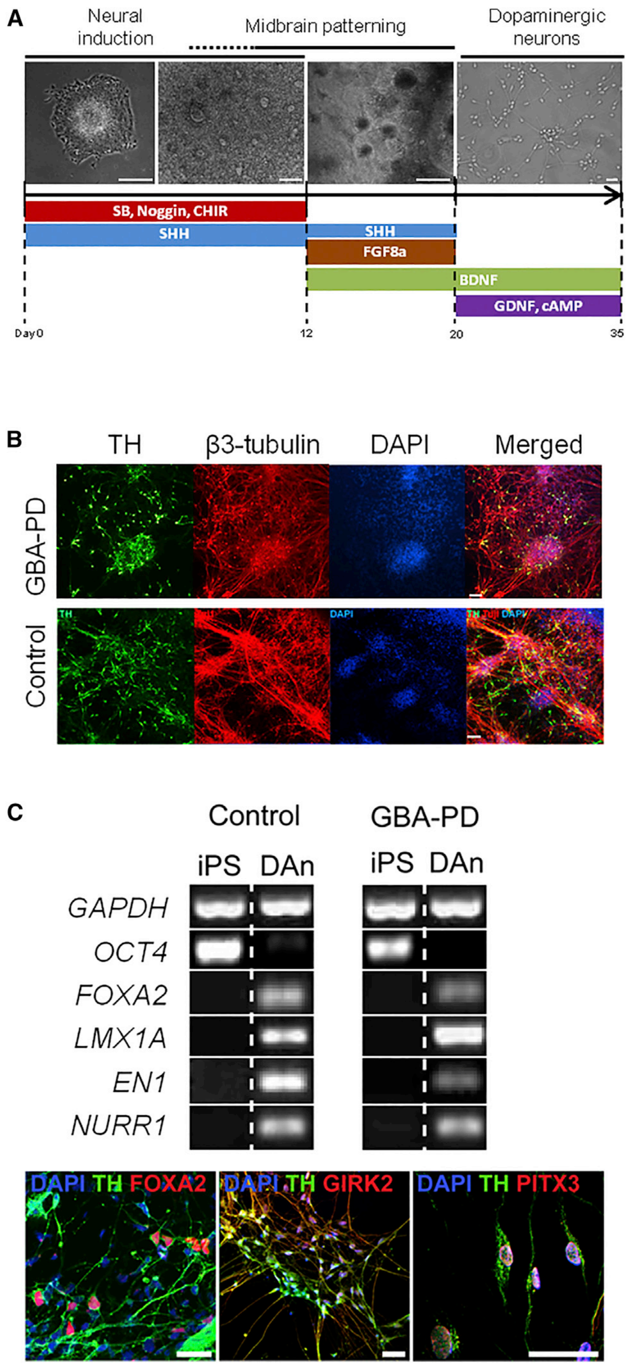

Figure 1. Generation and Characterization of iPSC-Derived Dopaminergic Neuronal Cultures

(A) Schematic overview of conditions used for differentiation of iPSCs into dopaminergic neuronal cultures, with bright field representations of cells at several stages of differentiation (scale bar, 50 μm).

(B) Immunohistochemistry representation of differentiated dopaminergic neuronal cultures at day 35 showing high expression levels for the neuronal marker β-3 tubulin (red), the dopaminergic neuron marker TH (green), and DAPI (blue).

(C) RT-PCR analysis showed reduced expression of the pluripotency marker OCT4 and increased expression of floor plate markers (FOXA2 and LMX1A) and mature dopaminergic markers (EN1 and NURR1) in differentiated dopaminergic neuronal cultures (DAn) versus undifferentiated (iPS). Immunofluorescence analyses also confirmed the co-expression of TH with FOXA1, GIRK2, and PITX3 (scale bar, 50 μm). Results are representative of at least three independent differentiation experiments performed in triplicate per cell line.
See also Figures S1–S3.

heterozygous *GBA-N370S* mutation on GCase protein level in the *GBA-N370S* PD lines. In addition to the expected 60 kDa isoform corresponding to mature GCase, we observed an extra isoform of apparently higher molecular weight only in dopaminergic neuronal cultures derived from *GBA-N370S* PD patients, which represented up to 50% of total GCase in these cultures (Figure 2A). This N370S-specific isoform was sensitive to treatment with EndoH, which yielded a third, lower molecular weight isoform (Figure 2B). This demonstrates that a proportion of GCase in the *N370S GBA* cell lines had retained high-mannose oligosaccharides, indicating failure to be processed in the Golgi, likely because of retention in the ER. In *GBA-N370S*-derived dopaminergic neuronal cultures, this EndoH-sensitive GCase isoform was significantly more abundant, representing around 20% of total GCase (Figure 2B). Samples were further treated with PNGase F to remove both high-mannose and complex N-linked glycans, and under these conditions no significant differences were observed in GCase protein levels between N370S mutant and control dopaminergic neuronal cultures (Figure 2C). Overall, these results indicate that the *GBA-N370S* mutation likely results in retention in the ER as a result of disrupting the structure and/or folding of the protein, without affecting the total amount of GCase.

LIMP2 is a GCase-specific lysosomal receptor (Reczek et al., 2007), which has been associated with PD risk (Do et al., 2011). In dopaminergic neuronal cultures derived from *GBA-N370S* PD patients, we observed increased LIMP2 protein expression compared with controls, although there was inter-individual variation between the *GBA-N370S* PD lines (Figure 2D). This result supports a role for LIMP2 in trafficking ER-retained mutant GCase from the ER to the lysosome (Reczek et al., 2007).

We next quantified the GCase substrate lipids in *GBA-N370S* PD dopaminergic cultures. No GlcCer accumulation was observed in patient cells, as measured by mass spectrometry quantification of individual species normalized to the ceramide precursor (Figure S4A). Notably, the distribution of GlcCer species was markedly different in *GBA-N370S* PD dopaminergic cultures compared with controls, with a 30% reduction for C20:0 GlcCer and a ~65% increase of C16:0 and C24:0 species (Figure 2E). Overall, these

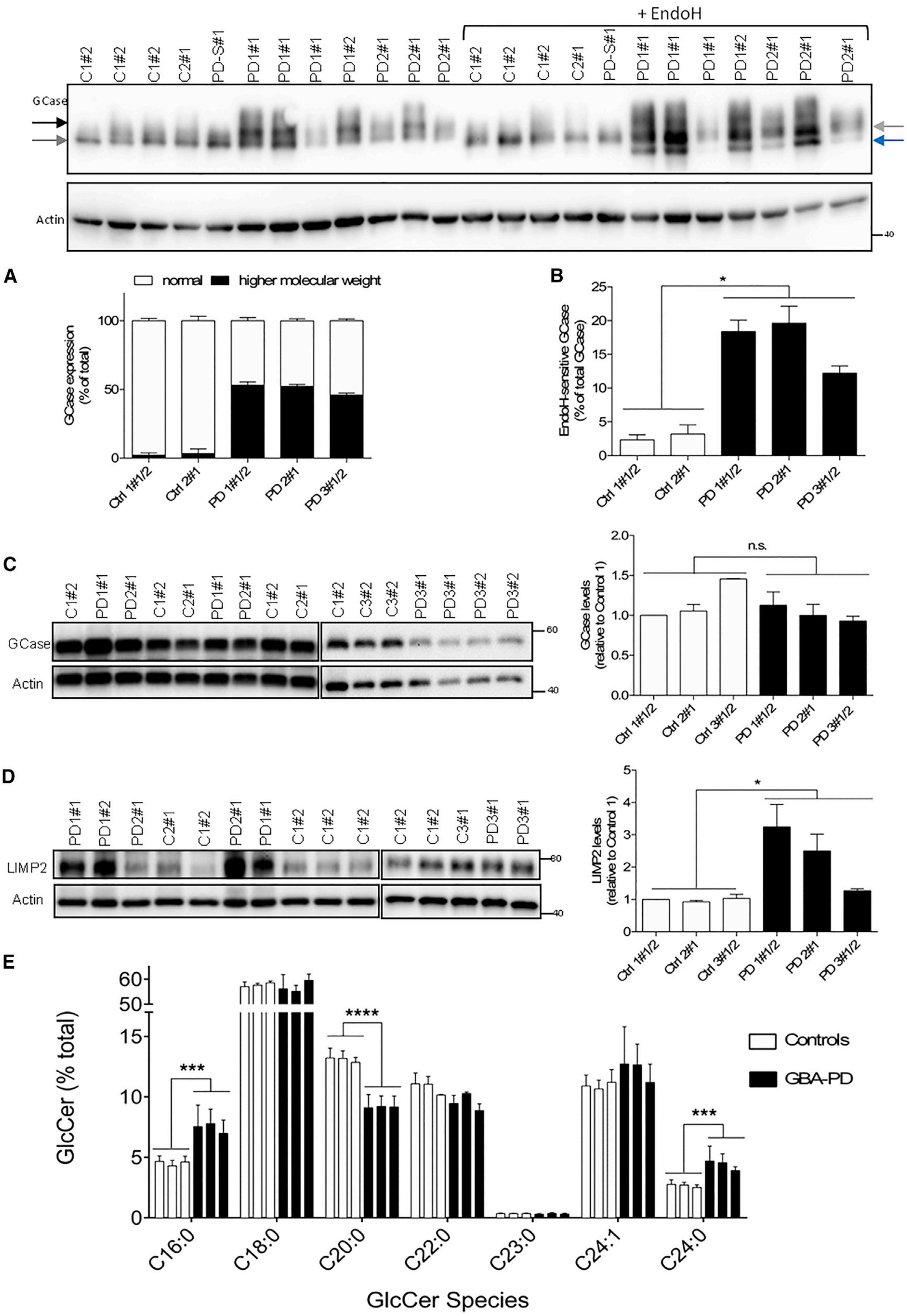

(legend on next page)

data indicate that the *GBA-N370S* heterozygous mutation results in abnormal lipid profiles not previously reported.

ER Stress and Autophagic Disturbances in *GBA-N370S* Dopaminergic Neuronal Cultures

Accumulation of misfolded proteins in the ER can overload the cellular capacity for protein re-folding and induce ER stress, leading to activation of the unfolded protein response (UPR) (Høyer-Hansen and Jäättelä, 2007). Analysis of the two ER-resident chaperones, Bip/GRP78 and calreticulin, revealed significant upregulation in *GBA-N370S* dopaminergic neuronal cultures when compared with control-derived dopaminergic cultures (Figure 3A). Upregulation of additional UPR mediators, PDI, calnexin, and IRE1alpha, confirmed UPR activation (Figures 3B–3D), although cleavage of *XBP1* mRNA was not observed (Figure S4B). *XBP1* splicing might not be a crucial transducer in this context as it has been previously detected in the brain in only one-third of *GBA* mutant PD patients (Gegg et al., 2012).

Since ER stress can result in autophagic disturbances (Yorimitsu et al., 2006) through the accumulation of misfolded proteins (Høyer-Hansen and Jäättelä, 2007), we examined the autophagosome content in our cultures by quantifying the levels of LC3B-II and LC3 puncta (Klionsky et al., 2012). LC3B-II, the lipidated form of the autophagosome marker LC3/Atg8, was significantly increased in *GBA-N370S*-derived dopaminergic neuronal cultures reflecting increased levels of autophagosomes (Figure 4A). This increase in LC3B-II was supported by immunofluorescence analysis that confirmed an increased number of $LC3^{+}$ puncta in dopamine neurons in *GBA-N370S* compared with controls (Figure 4B). Furthermore, beclin1, a key regulator of autophagy used to study autophagosome formation, was found increased in *GBA-N370S* dopaminergic neuronal cultures from two patients, consistent with an autophagic perturbation (Figure 4C).

Impaired Lysosomal Degradation in *GBA-N370S* Dopaminergic Neurons

Accumulation of autophagosomes can also reflect a defect in lysosomal clearance (Klionsky et al., 2012). Since the *GBA-N370S* mutation is located in the catalytic domain of GCase (Wei et al., 2011), we determined the impact of the heterozygous *GBA-N370S* mutation on the activity of this lysosomal enzyme. Dopaminergic neuronal cultures from *GBA-N370S* PD patients had significantly reduced GCase activity (~50% decrease) when compared with control individuals (Figure 5A). Since reduced GCase activity could impair the overall lysosomal degradation capacity of dopamine neurons, we analyzed the levels of p62/SQSTM1 that reflect autophagic-mediated degradation (Klionsky et al., 2012). p62 protein levels were increased specifically in *GBA-N370S* dopaminergic neuronal cultures (Figure 5B), which supports a defect of these cells in the clearance of autophagic vacuoles. To follow up the findings from *GBA-N370S* cultures, we next compared *GBA-N370S* and control dopamine neurons for ultrastructural alterations by electron microscopy (EM), specifically in identified TH-positive neurons. We found an accumulation of electron-dense debris within lysosomal structures in *GBA-N370S* mutant dopaminergic neurons in TH immunogold-labeled cells, (Figures 5C–5E), which likely represents undegraded cargo found in *GBA-N370S* dopamine neurons.

Enlarged Lysosomal Compartment in *GBA-N370S* Dopaminergic Neurons

Following the previous observations suggesting impaired lysosomal degradation, the lysosomal compartment was analyzed in more detail. Dopaminergic neuronal cultures derived from *GBA-N370S* PD patients showed increased expression of the lysosomal markers LAMP1 (Figure 6A) and LAMP2A (Figure 6B) when compared with cultures derived from controls. The levels of cathepsin D, a major

Figure 2. Aberrant GCase Post-translation Modification in Heterozygous *GBA-N370S* PD Dopaminergic Neuronal Cultures
Representative western blot for differentiated dopaminergic neuronal cultures demonstrates the presence of two isoforms (gray and black arrows) for GCase protein specific for *GBA-N370S* cultures.
(A) Quantification of GCase isoforms by western blot (as percentage of total GCase) confirms the higher molecular weight isoform (black bars) to be specific to *GBA-N370S* dopaminergic neuronal cultures, representing up to 50% of total GCase levels.
(B) The GCase top isoform was sensitive to EndoH treatment shifting to a lower size band (blue arrow). Quantification of the EndoH-sensitive band represented as a percentage of total GCase for the respective line. Student's t test; *p < 0.0001. PD-S#1 corresponds to a sporadic PD sample run but not analyzed further in this study.
(C) No change of total GCase expression levels after PNGase F treatment. Representative western blot and quantification of GCase is shown. Student's t test; not significant.
(D) Increased LIMP2 protein levels in heterozygous *GBA-N370S* PD dopaminergic cultures. Student's t test; *p < 0.005. In each case, data represent the mean ± SEM from performing at least three independent differentiation experiments per line each analyzed in triplicate.
(E) Analysis of different GlcCer species (C16:0, C18:0, C20:0, C22:0, C23:0, C24:0, C24:1, C24:0) as a percentage of total GlcCer shows a difference in the distribution of GlcCer species in *GBA-N370S* PD dopaminergic cultures compared with controls. Each bar represents the mean ± SEM of independent differentiated lines: three control and three *GBA-N370S* PD done in triplicate (n = 3). Student's t test; ***p < 0.005; ****p < 0.0001.
See also Figure S4A.

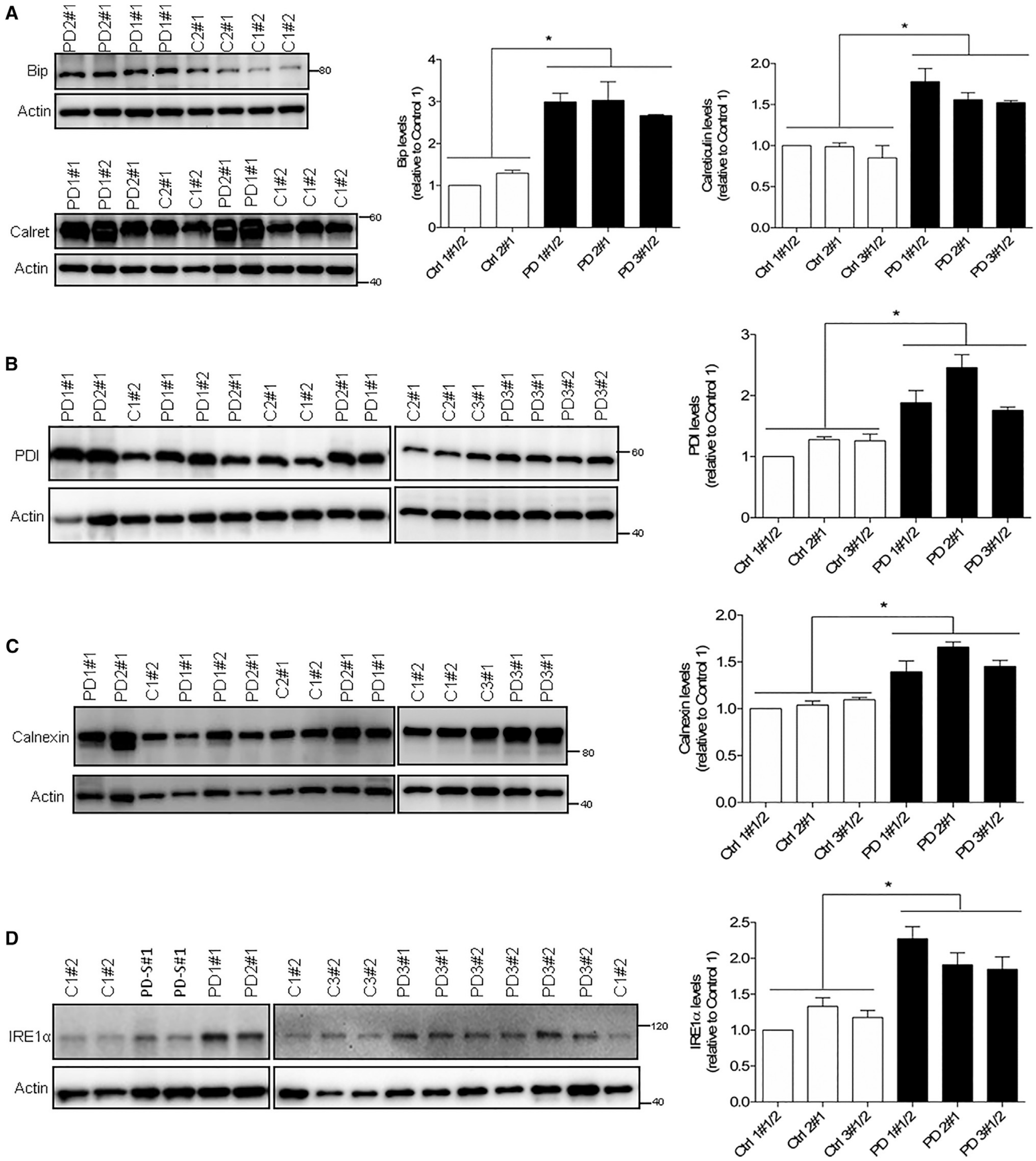

Figure 3. ER Stress Upregulation in Heterozygous *GBA-N370S* Dopaminergic Neuronal Cultures

(A) Representative western blot and quantification shows increased protein levels of the ER chaperones Bip and calreticulin.

(B–D) Representative western blot and quantification of expression of further ER stress markers, namely PDI (B), calnexin (C), and IRE1α (D).

In each case, data represent the mean ± SEM from performing at least three independent differentiation experiments per line each analyzed in triplicate. Student's t test; *$p < 0.0001$. PD-S#1 corresponds to a sporadic PD sample run but not analyzed further in this study. See also Figure S4B.

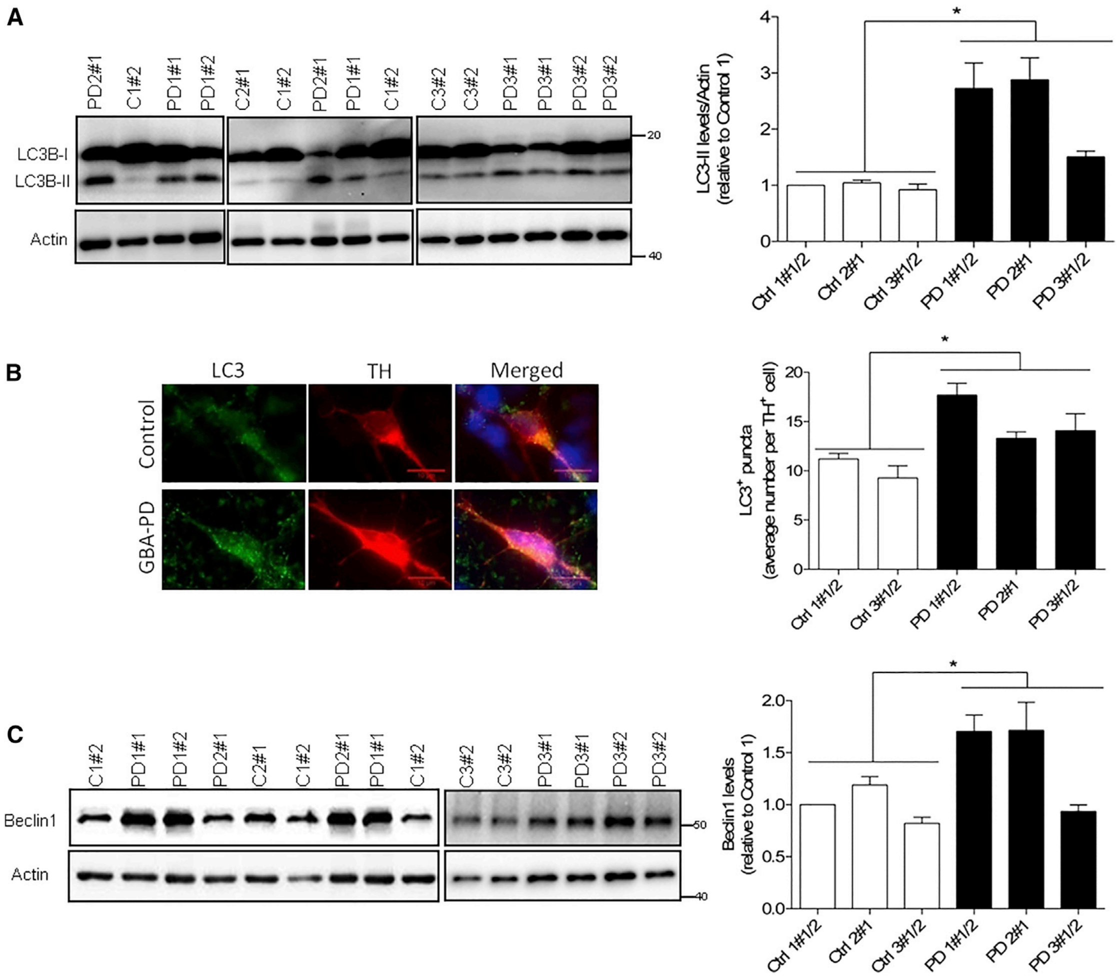

Figure 4. Autophagic Disturbances in Heterozygous *GBA-N370S* PD Dopaminergic Neuronal Cultures

(A) Representative western blot analysis and quantification showing increased LC3B-II protein levels. Student's t test; $*p < 0.0001$.

(B) Increased number of $LC3^+$ puncta in TH^+ cells. Immunofluorescence staining for LC3B (green) in dopaminergic TH-positive neurons (red) in control and PD patient dopaminergic neuronal cultures (scale bar, 20 μm). Student's t test; $*p < 0.0001$.

(C) Representative western blot and quantification of Beclin1 protein levels in differentiated dopaminergic cultures. Student's t test; $*p < 0.05$. In each case, data represent the mean ± SEM from performing at least three independent differentiation experiments per line each analyzed in triplicate.

lysosomal enzyme involved in α-synuclein degradation (Sevlever et al., 2008) and associated with GCase deficiency (Vitner et al., 2010), was also found increased in dopaminergic neuronal cultures from *GBA-N370S* patients (Figure 6C).

The enlarged lysosomal compartment was confirmed by EM, which demonstrated a ~2-fold increase in the number of lysosomes in TH immunogold-labeled cells (Figures 6D and 6E). Furthermore, the area occupied by lysosomal organelles within TH-positive neurons detected by EM was increased by ~2.5-fold in *GBA-N370S* cells compared with controls (Figures 6D and 6F), suggesting an enlargement of the lysosomal compartment. EM data also highlighted that the enlargement of the lysosomal compartment was specific to TH-positive dopaminergic neurons, as no differences were found in TH-negative cells (Figures S5A and S5B), emphasizing the importance of studying a disease-relevant cell type. The enlarged lysosomal compartment may reflect impaired lysosomal degradation capacity in dopamine neurons, which was not altered in TH-negative cells. *GBA-N370S* dopaminergic cultures were still capable of responding efficiently to situations of increased autophagic demand, as demonstrated by increased lysosomal numbers under starvation conditions determined by EM analysis (Figure S5C). Treatment with the V-ATPase inhibitor bafilomycin A1 also resulted in increased number of lysosomes in TH-positive neurons, which indicates that the process of lysosomal

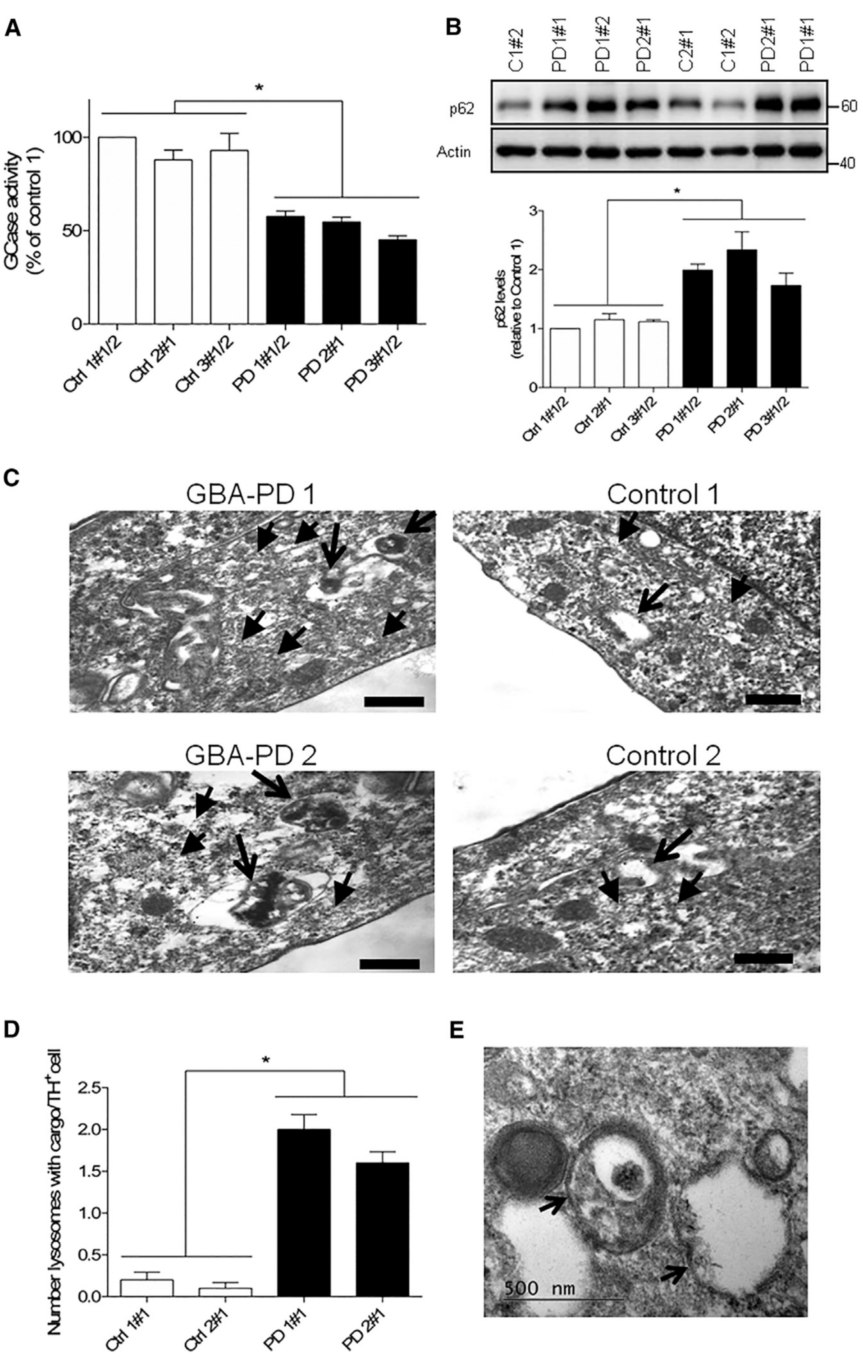

Figure 5. Impaired Lysosomal Degradation Capacity in Heterozygous *GBA-N370S* PD Dopamine Neurons

(A) GCase enzyme activity in differentiated dopaminergic neuronal cultures was reduced to 50% when compared with controls. Student's t test; $*p < 0.0001$.

(B) Increased p62 protein levels in heterozygous *GBA-N370S* dopaminergic cultures when compared with controls. Representative western blot analysis and respective quantification. In each case, data represent the mean ± SEM from performing at least three independent differentiation experiments per line each analyzed in triplicate. Student's t test; $*p < 0.0001$.

(C) Electron micrographs showing representative examples of undegraded cargo observed within lysosomal structures in TH immunogold-positive *GBA-N370S*-derived neurons. Arrows indicate LAMP1 positive structures of the lysosomal pathway; arrowheads indicate TH immunogold (5 nm). Scale bar, 200 nm.

(D) Quantification shows increased number of lysosomes with undegraded cargo per TH^+ cell in *GBA-N370S* cultures compared with controls. Data represent the mean ± SEM of $n = 20$ cells per line from two independent differentiation experiments. Student's t test; $*p < 0.001$.

(E) A high magnification image reveals double-membrane vesicles corresponding to autophagolysosomes, with arrows indicating double membranes.

biogenesis is still inducible in *GBA-N370S* dopaminergic cultures (Figure S5C).

Increased Extracellular α-Synuclein in *GBA-N370S* Dopaminergic Neuronal Cultures

Accumulation of α-synuclein is the characteristic pathological marker of PD and can result from defective cellular clearance mechanisms. α-Synuclein is degraded by multiple pathways including autophagy and the autophagy-lysosome pathway (Cuervo et al., 2004), and α-synuclein levels have been shown to be altered in response to pharmacologically induced autophagic impairments (Klucken et al., 2012) and ER stress (Hoepken et al., 2008). Analysis of the intracellular α-synuclein content in dopaminergic neuronal cultures from *GBA-N370S* PD showed no differences when compared with controls (Figure 7A). However, recent reports have shown that impairments of the autophagic machinery can affect the release of α-synuclein in

neuroblastoma cells, rat primary neurons, and mouse fibroblasts (Alvarez-Erviti et al., 2011; Ejlerskov et al., 2013; Emmanouilidou et al., 2010; Jang et al., 2010; Lee et al., 2013). We therefore investigated if the autophagic/lysosomal disturbances observed in *GBA-N370S* dopaminergic neuronal cultures could alter α-synuclein release in our human PD-specific cell model. Analysis by ELISA revealed the presence of α-synuclein in the culture media of differentiated dopaminergic cultures, which increased over time (Figure 7B). Importantly, the levels of α-synuclein in the media were found to be increased for mature dopaminergic neuronal cultures differentiated from *GBA-N370S* patients when compared with controls (Figure 7C), suggesting a possible link between the autophagic/lysosomal disturbances we report and the modulation of α-synuclein release.

The secretion of α-synuclein has been associated with exosomes in α-synuclein overexpressing neuroblastoma cells, following an unconventional secretory pathway (Emmanouilidou et al., 2010). We therefore examined if a similar mechanism could be associated with α-synuclein release in our PD patient model. Exosomes were isolated from dopaminergic neuronal cultures and analyzed by nanoparticle tracking analysis. This analysis did not reveal any significant difference in the nodal size of exosomes between control and *GBA-N370S* cultures (Figure S6). In addition, there was no difference in the concentration of exosomes secreted by control or *GBA-N370S* dopaminergic cultures (Figure S6). Western blot analysis of extracted exosomes confirmed the detection of generic (CD81) and neuron-specific (L1CAM) exosomal markers, and the absence of calnexin, an indicator of apoptotic bodies (Figure 7D). These data indicate that extracellular α-synuclein is not associated with neuron-derived exosomes. Accordingly, ELISA analysis showed that exosome depletion of conditioned media did not change the α-synuclein levels in the media and it was estimated that exosome-associated α-synuclein constitutes less than about 1.6% of the total extracellular fraction.

To further investigate the mechanisms resulting in α-synuclein release in dopaminergic cultures carrying *GBA-N370S* mutation, cells were treated with either chloroquine (CQ) or with bafilomycin A1, both of which cause impaired lysosomal function by neutralizing lysosomal pH (Juhasz, 2012). Each treatment resulted in a significant increase in released α-synuclein in *GBA-N370S* dopaminergic cultures (Figure 7E). Both treatments had a bigger impact on patient cells than controls, suggesting a role for lysosomal function in α-synuclein release in *GBA-N370S* dopaminergic cultures. To explore a possible role for the ER/Golgi-dependent classical export pathway in α-synuclein release, cultures were treated with brefeldin A, which blocks the trafficking between ER and Golgi. Brefeldin A treatment resulted in increased release of α-synuclein in all dopaminergic cultures (Figure 7E), more pronounced in *GBA-N370S* lines, suggesting that the ER/Golgi vesicle-mediated transport is also involved in α-synuclein release. Overall, these data confirm an increased release of α-synuclein in *GBA-N370S* PD dopaminergic neuronal cultures associated with an impairment of the autophagic/lysosome pathway, offering a possible therapeutic target.

DISCUSSION

Using human iPSCs derived from PD patients carrying the heterozygous *GBA-N370S* mutation, we have identified relevant mechanisms by which a heterozygous *GBA* mutation associated with PD may increase cellular susceptibility to disease. Our results highlight an important role of heterozygous mutant GCase in the disruption of protein homeostasis in dopaminergic neurons, ultimately leading to increased α-synuclein release, which may be central for the early pathogenesis of PD. Differentiation of ten independent iPSC lines from three controls and three unrelated *GBA-N370S* PD patients into dopaminergic neurons allowed us to robustly investigate this genetic contribution in an appropriate human model of early PD pathology.

Our data suggest that the heterozygous *GBA-N370S* mutation results in the retention of GCase within the ER in PD iPSC-derived dopaminergic neuronal cultures. We found no evidence of a reduction of GCase protein levels in our multiple lines derived from three unrelated patients, indicating that the protein is not targeted for degradation. We have observed an overall increased expression of LIMP2, a GCase-specific lysosomal receptor, in dopaminergic cultures from *GBA-N370S* patients. Increased LIMP2 expression is likely to be part of a cellular response to *GBA* mutations, as it has been previously shown that LIMP2 overexpression increases transportation of ER-retained GCase toward the lysosomes (Reczek et al., 2007).

We observed the upregulation of multiple ER stress markers in *GBA-N370S*-derived dopaminergic neuronal cultures, which likely reflects a dysregulation of the ER homeostatic environment induced by misprocessing of misfolded GCase similar to a postmortem analysis of PD *GBA* patients showing UPR activation (Gegg et al., 2012).

We have also identified alterations in the autophagy pathway in our PD model. Levels of autophagosomes were increased in dopamine neurons from *GBA-N370S* lines, and we provide multiple lines of evidence to support an impairment of general autophagic vacuole clearance. In addition, multiple lysosomal markers were upregulated in *GBA-N370S* dopaminergic lines consistent with an impairment of lysosomal degradation capacity. Moreover, an enlargement of the lysosomal compartment was identified, specifically within TH immunogold-identified

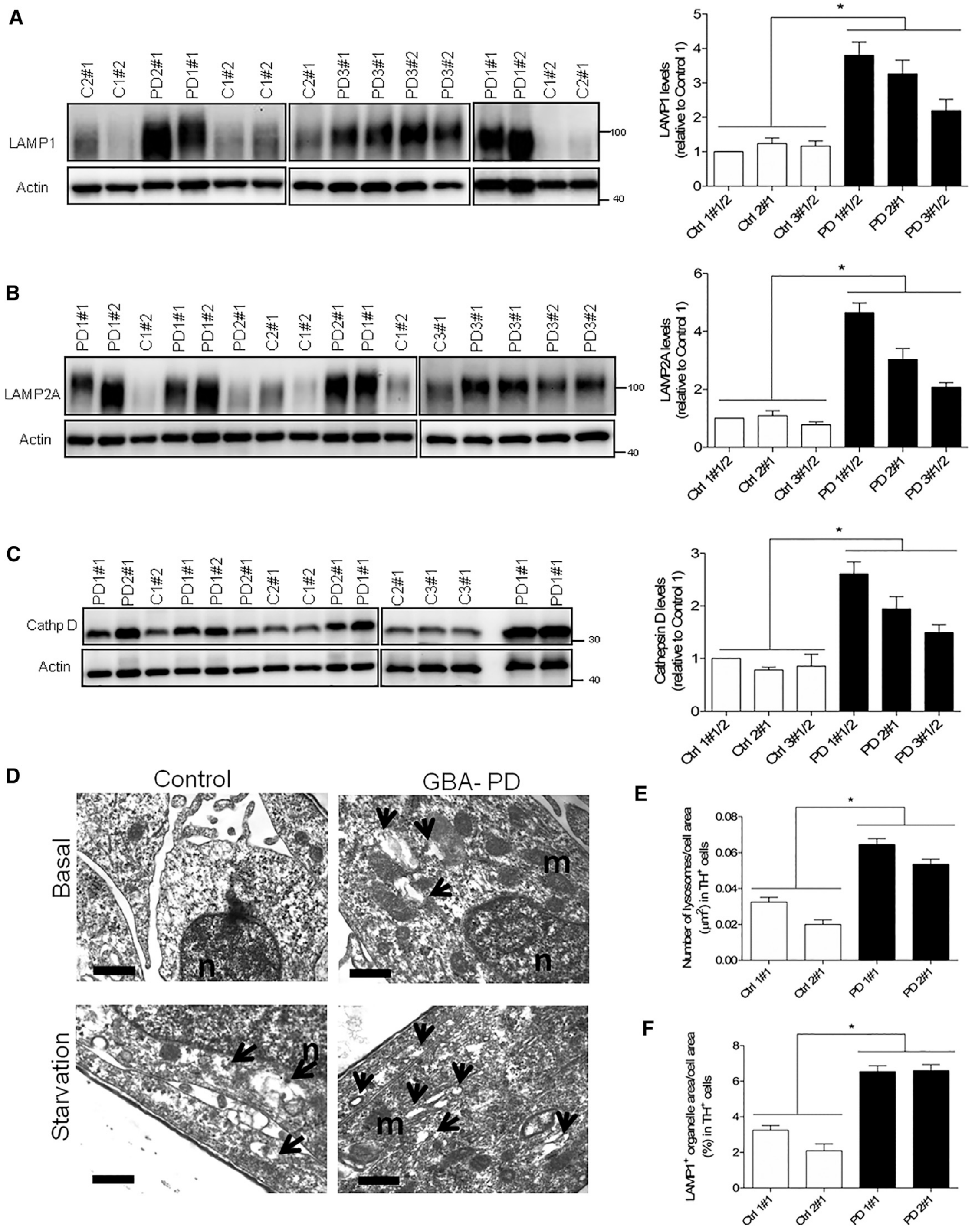

Figure 6. Enlargement of the Lysosomal Compartment in Dopamine Neurons Derived from PD Patients Carrying a Heterozygous *GBA-N370S* Mutation

(A–C) Increased protein levels of multiple lysosomal markers in heterozygous *GBA-N370S* PD dopaminergic cultures. Representative western blots and quantifications for the expression of: (A) LAMP1, (B) LAMP2A, and (C) cathepsin D. In each case, data represent the mean ± SEM from performing at least three independent differentiation experiments per line each analyzed in triplicate. Student's t test; $^*p < 0.001$.
(D) Electron micrographs showing representative images of immunogold-labeled TH-positive neurons for differentiated controls and *GBA-N370S* neurons in basal and under starvation conditions. Arrows indicate LAMP1-positive structures of the lysosomal pathway. Scale bar, 200 nm.

(legend continued on next page)

dopaminergic neurons derived from patient's lines, characterized by increased number and size of lysosomal structures. Lysosomal dysfunction is a key pathological event in PD (Dehay et al., 2010), and the specificity to dopamine neurons may explain the preferential vulnerability of these neurons.

Altered cellular lipid composition can be associated with ER disturbances (Basseri and Austin, 2012) and autophagic impairments (Koga et al., 2010). Although the mechanisms underlying cellular distribution of GlcCer species in neurons is not well understood, lipid contents are known to differ in membranes undergoing ER-Golgi maturation or fusion within various organelles (Halter et al., 2007). The different GlcCer species distribution observed in our neuronal cultures between control and *GBA-N370S* lines may be due to the different trafficking of GCase. As a result of altered trafficking, the mutant GCase enzyme may hydrolyze a species that might not have been degraded in normal cells, thereby decreasing total amounts of certain lipids (e.g., 30% reduction of C20:0 GlcCer levels in *GBA-N370S* neurons). On the other hand, reduced GCase concentration in particular sub-compartments may promote the accumulation of other species (e.g., ~65% increase in C16:0 and C24:0 species) in *GBA-N370S* neurons.

It is known that α-synuclein is degraded in part by the autophagic/lysosomal pathway (Cuervo et al., 2004). We observed no differences for the intracellular α-synuclein levels in dopaminergic cultures derived from three patients carrying the *GBA-N370S* mutation, which is in line with the report of Schöndorf et al. (2014). Although postmortem studies have suggested the possibility of increased intracellular α-synuclein in brains from PD patients carrying GBA mutations, postmortem tissue likely reflects a much more advanced stage of disease pathology (Choi et al., 2012; Gegg et al., 2012).

Since recent reports have demonstrated that perturbations of the autophagic pathway can affect α-synuclein release in neuroblastoma cells and mouse fibroblasts, we decided to investigate if a similar mechanism operated in our physiological human model of PD. Interestingly, we observed increased levels of α-synuclein released in the culture media from *GBA-N370S* dopaminergic neuronal cultures, which may be a consequence of the autophagic/lysosomal defects in these cells. To further investigate the potential mechanisms by which *GBA-N370S* mutation results in increased α-synuclein release, dopaminergic cultures were treated with the lysosomal function inhibitors CQ or bafilomycin A1. Each treatment increased the levels of released α-synuclein, further suggesting the involvement of the lysosome in the release of α-synuclein in *GBA-N370S* dopaminergic cultures. These results suggest that the autophagic and lysosomal deficits we describe result in increased α-synuclein release in *GBA-N370S* dopaminergic cultures. The lack of significant increase in released α-synuclein in control cultures after induced lysosomal dysfunction suggests a difference in the way the lysosomes handle α-synuclein between PD and control cultures. In addition, although earlier reports showed that α-synuclein release was independent of the ER/Golgi classical export pathway (Emmanouilidou et al., 2010; Jang et al., 2010; Lee et al., 2005), recent studies have shown that in some models, including enteric neurons, α-synuclein release is dependent on ER/Golgi exocytosis (Chutna et al., 2014; Paillusson et al., 2013). Our results indicate that α-synuclein release is also sensitive to brefeldin A treatment, supporting a role for the ER/Golgi classical pathway in the release of α-synuclein in both *GBA-N370S* and control dopaminergic cultures. Taken together, our data suggest that α-synuclein release can occur via both conventional and unconventional pathways in dopaminergic neuronal cultures from *GBA-N370S* cells.

α-Synuclein release could be an important early event in the pathology of PD whereby uptake of α-synuclein by neighboring neurons could result in impaired cellular proteostasis (Desplats et al., 2009). α-Synuclein involvement in this context might be complex since it was also previously shown to cause ER stress, block ER-Golgi trafficking (Cooper et al., 2006), induce lysosomal rupture (Freeman et al., 2013), decrease lysosomal function (Cuervo et al., 2004), and inhibit GCase activity (Mazzulli et al., 2011).

Secreted α-synuclein has also been associated with exosomes in the context of neuroblastoma cells overexpressing α-synuclein (Alvarez-Erviti et al., 2011; Emmanouilidou et al., 2010). To expand on the observation of increased α-synuclein release in our model, we extracted and analyzed human iPSC-derived neuronal exosomes. We did not find a readily detectable association of α-synuclein with exosomes in our model, which is consistent with our recent proteomic analysis in serum-derived microvesicles from PD patients (Tomlinson et al., 2015).

Heterozygous *GBA-N370S* mutations are the strongest risk factor for PD and have also been associated with related

(E) Quantification of EM data shows an increase number of lysosomes in basal conditions for TH neurons in *GBA-N370S* cultures compared with controls.
(F) The lysosomal compartment, represented as a percentage of the cell area occupied by LAMP1-labeled organelles, was also increased in size in TH-positive neurons in basal conditions in *GBA-N370S* cultures. In each case, data represent the mean ± SEM of n = 20 cells per line from two independent differentiation experiments. Student's t test; *p < 0.001.
See also Figure S5.

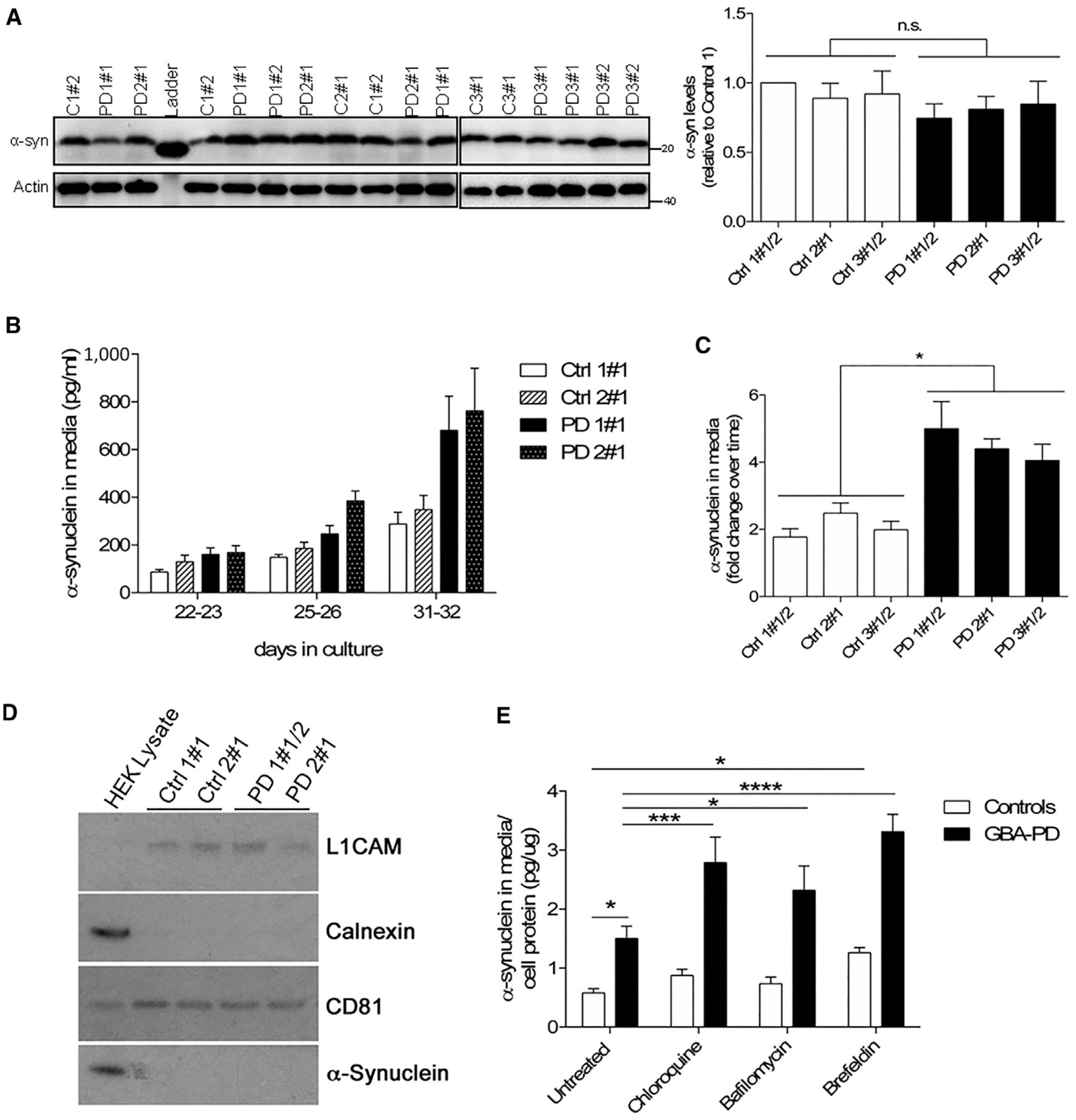

Figure 7. Increased Extracellular α-Synuclein in Heterozygous *GBA-N370S*-Derived Dopaminergic Neuronal Cultures

(A) There is no difference in the intracellular α-synuclein content in dopaminergic neuronal cultures from controls and *GBA-N370S* PD lines. Representative western blot and quantification for intracellular α-synuclein levels.

(B) Analysis of culture media by ELISA revealed the presence of α-synuclein, which increased over time.

(C) Levels of extracellular α-synuclein in culture media were higher for heterozygous *GBA-N370S* dopaminergic cultures when compared with controls, during maturation, measured by ELISA. Fold change of neurons at day 31–36 compared with early neurons at day 21. In each case, data represent the mean ± SEM from performing at least three independent differentiation experiments per line each analyzed in triplicate. Student's t test; *$p < 0.001$.

(D) Western blot analysis of culture media extracted exosomes confirmed the expression of CD81 and L1CAM exosomal markers in the absence of calnexin. Data represent the mean of replicates ± SEM from performing at least three independent differentiation experiments per line.

(E) Modulation of α-synuclein release in differentiated dopaminergic neurons from control and *GBA-N370S* dopaminergic cultures by treatment with CQ, bafilomycin A1, and brefeldin A, measured by the MesoScale Discovery platform. Each bar represents the mean ± SEM of differentiated lines from at least three independent individuals done in duplicate (n = 6–8). Two-way ANOVA; *$p < 0.05$; ***$p < 0.005$; ****$p < 0.0001$.

See also Figure S6.

Lewy body disorders, in particular with dementia with Lewy bodies, where they represent an important risk factor (Nalls et al., 2013). Dopaminergic neurons of the substantia nigra are lost in both PD and dementia with Lewy bodies, which may occur through the mechanisms of altered protein homeostasis we describe here. Interestingly, although a *GBA* mutation increases the relative risk for PD by 30-fold compared with the general population (McNeill et al., 2012), most *GBA* mutation carriers do not develop disease. This suggests that a *GBA* mutation confers strong susceptibility, but that a second hit, likely genetic or environmental, is required to drive pathology. Pharmacological rescue in PD *GBA-N370S* neurons using chemical chaperones such as isofagomine and ambroxol, or treatment with recombinant GCase, will allow further dissection of the mechanisms by which GBA mutations confer susceptibility to PD and may identify promising protective therapies.

Taken together, our findings suggest that in PD, the heterozygous *GBA-N370S* mutation leads to the misprocessing of GCase, ER stress upregulation, and autophagic/lysosomal dysfunction in dopaminergic neurons. We propose that this combination of disturbances impairs protein homeostasis in dopamine neurons, which leads to increased α-synuclein release. Such cellular events in combination may lead to preferential dopamine neuronal vulnerability in PD.

EXPERIMENTAL PROCEDURES

Participant Recruitment

Participants were recruited to this study having given signed informed consent, which included mutation screening and derivation of hiPSC lines from skin biopsies (Ethics Committee: National Health Service, Health Research Authority, NRES Committee South Central, Berkshire, UK, who specifically approved this part of the study (REC 10/H0505/71).

Culture and Reprogramming of Primary Fibroblasts

Skin punch biopsies (4 mm in diameter) were obtained from participants and low passage fibroblast cultures established and transduced with reprogramming retroviruses (c-MYC, KLF4, SOX2, OCT3/4, and Nanog). Colonies displaying iPSC morphology were picked on day 28 and passaged on murine embryonic fibroblasts by manual dissection every 5–7 days. Full characterization information from the PD patient lines is given in the Supplemental Experimental Procedures. The control iPSC lines have been described fully elsewhere (Hartfield et al., 2014; van Wilgenburg et al., 2013).

RT-PCR, Immunocytochemistry, and Western Blot Analysis

These procedures were performed using standard methods and details are given in Supplemental Experimental Procedures. Representative western blots for antibodies used are shown Figure S7.

α-Synuclein Measurements

An in-house ELISA for the accurate quantification of α-synuclein concentration was developed by using two commercially available α-synuclein-specific antibodies: the monoclonal Syn-1 (BD Transductions) as the capture antibody and the polyclonal C-20 (Santa Cruz) as the detection antibody, which was used after its covalent conjugation with horseradish peroxidase (Emmanouilidou et al., 2011). MesoScale Discovery assays were used following the manufacturer's instructions. Further details are given in the Supplemental Experimental Procedures.

ACCESSION NUMBERS

The SNP datasets and the Illumina HT12v4 transcriptome array results have been deposited in Gene Expression Omnibus under accession number GEO: GSE53426.

SUPPLEMENTAL INFORMATION

Supplemental Information includes Supplemental Experimental Procedures, seven figures, and one table and can be found with this article online at http://dx.doi.org/10.1016/j.stemcr.2016.01.013.

ACKNOWLEDGMENTS

The work was supported by the Monument Trust Discovery Award from Parkinson's UK, The Oxford Martin School, which provides core support to the James Martin Stem Cell Facility (LC0910-004) and the Innovative Medicines Initiative Joint Undertaking under grant agreement number 115439, resources of which are composed of financial contribution from the European Union's Seventh Framework Program (FP7/2007e2013) and EFPIA companies' in kind contribution. The work was supported by the Monument Trust Discovery Award from Parkinson's UK. The OPDC Discovery cohort was also supported by the National Institute for Health Research (NIHR) Oxford Biomedical Research Centre based at Oxford University Hospitals NHS Trust and University of Oxford, and the Dementias and Neurodegenerative Diseases Research Network (DeNDRoN). H.J.R.F. was supported by a Fundacao para a Ciencia e Tecnologia (FCT) grant (SFRH/BD/65787/2009) and S.C. has been supported by a Wellcome Trust Career Re-Entry Fellowship (WT082260/Z/07/Z). We thank the High-Throughput Genomics Group at the Wellcome Trust Centre for Human Genetics, Oxford (funded by Wellcome Trust grant reference 090532/Z/09/Z and MRC Hub grant G0900747 91070) for the generation of Illumina genotyping and transcriptome data.

Received: August 13, 2015
Revised: January 15, 2016
Accepted: January 18, 2016
Published: February 18, 2016

REFERENCES

Alegre-Abarrategui, J., Christian, H., Lufino, M.M., Mutihac, R., Venda, L.L., Ansorge, O., and Wade-Martins, R. (2009). LRRK2 regulates autophagic activity and localizes to specific membrane

microdomains in a novel human genomic reporter cellular model. Hum. Mol. Genet. *18*, 4022–4034.

Alvarez-Erviti, L., Seow, Y., Schapira, A.H., Gardiner, C., Sargent, I.L., Wood, M.J., and Cooper, J.M. (2011). Lysosomal dysfunction increases exosome-mediated alpha-synuclein release and transmission. Neurobiol. Dis. *42*, 360–367.

Basseri, S., and Austin, R.C. (2012). Endoplasmic reticulum stress and lipid metabolism: mechanisms and therapeutic potential. Biochem. Res. Int. *2012*, 841362.

Choi, J.M., Kim, W.C., Lyoo, C.H., Kang, S.Y., Lee, P.H., Baik, J.S., Koh, S.B., Ma, H.I., Sohn, Y.H., Lee, M.S., et al. (2012). Association of mutations in the glucocerebrosidase gene with Parkinson disease in a Korean population. Neurosci. Lett. *514*, 12–15.

Chutna, O., Goncalves, S., Villar-Pique, A., Guerreiro, P., Marijanovic, Z., Mendes, T., Ramalho, J., Emmanouilidou, E., Ventura, S., Klucken, J., et al. (2014). The small GTPase Rab11 co-localizes with alpha-synuclein in intracellular inclusions and modulates its aggregation, secretion and toxicity. Hum. Mol. Genet. *23*, 6732–6745.

Cooper, A.A., Gitler, A.D., Cashikar, A., Haynes, C.M., Hill, K.J., Bhullar, B., Liu, K., Xu, K., Strathearn, K.E., Liu, F., et al. (2006). Alpha-synuclein blocks ER-Golgi traffic and Rab1 rescues neuron loss in Parkinson's models. Science *313*, 324–328.

Cuervo, A.M., Stefanis, L., Fredenburg, R., Lansbury, P.T., and Sulzer, D. (2004). Impaired degradation of mutant alpha-synuclein by chaperone-mediated autophagy. Science *305*, 1292–1295.

Dehay, B., Bové, J., Rodríguez-Muela, N., Perier, C., Recasens, A., Boya, P., and Vila, M. (2010). Pathogenic lysosomal depletion in Parkinson's disease. J. Neurosci. *30*, 12535–12544.

Desplats, P., Lee, H.-J., Bae, E.-J., Patrick, C., Rockenstein, E., Crews, L., Spencer, B., Masliah, E., and Lee, S.-J. (2009). Inclusion formation and neuronal cell death through neuron-to-neuron transmission of alpha-synuclein. Proc. Natl. Acad. Sci. USA *106*, 13010–13015.

Do, C.B., Tung, J.Y., Dorfman, E., Kiefer, A.K., Drabant, E.M., Francke, U., Mountain, J.L., Goldman, S.M., Tanner, C.M., Langston, J.W., et al. (2011). Web-based genome-wide association study identifies two novel loci and a substantial genetic component for Parkinson's disease. PLoS Genet. *7*, e1002141.

Ejlerskov, P., Rasmussen, I., Nielsen, T.T., Bergström, A.-L., Tohyama, Y., Jensen, P.H., and Vilhardt, F. (2013). Tubulin polymerization-promoting protein (TPPP/p25α) promotes unconventional secretion of α-synuclein through exophagy by impairing autophagosome-lysosome fusion. J. Biol. Chem. *288*, 17313–17335.

Emmanouilidou, E., Melachroinou, K., Roumeliotis, T., Garbis, S.D., Ntzouni, M., Margaritis, L.H., Stefanis, L., and Vekrellis, K. (2010). Cell-produced alpha-synuclein is secreted in a calcium-dependent manner by exosomes and impacts neuronal survival. J. Neurosci. *30*, 6838–6851.

Emmanouilidou, E., Elenis, D., Papasilekas, T., Stranjalis, G., Gerozissis, K., Ioannou, P.C., and Vekrellis, K. (2011). Assessment of α-synuclein secretion in mouse and human brain parenchyma. PLoS One *6*, e22225.

Freeman, D., Cedillos, R., Choyke, S., Lukic, Z., McGuire, K., Marvin, S., Burrage, A.M., Sudholt, S., Rana, A., O'Connor, C., et al. (2013). Alpha-synuclein induces lysosomal rupture and cathepsin dependent reactive oxygen species following endocytosis. PLoS One *8*, e62143.

Gegg, M.E., Burke, D., Heales, S.J.R., Cooper, J.M., Hardy, J., Wood, N.W., and Schapira, A.H.V. (2012). Glucocerebrosidase deficiency in substantia nigra of parkinson disease brains. Ann. Neurol. *72*, 455–463.

Goker-Alpan, O., Schiffmann, R., LaMarca, M.E., Nussbaum, R.L., McInerney-Leo, A., and Sidransky, E. (2004). Parkinsonism among Gaucher disease carriers. J. Med. Genet. *41*, 937–940.

Goker-Alpan, O., Giasson, B.I., Eblan, M.J., Nguyen, J., Hurtig, H.I., Lee, V.M., Trojanowski, J.Q., and Sidransky, E. (2006). Glucocerebrosidase mutations are an important risk factor for Lewy body disorders. Neurology *67*, 908–910.

Halter, D., Neumann, S., van Dijk, S.M., Wolthoorn, J., de Maziere, A.M., Vieira, O.V., Mattjus, P., Klumperman, J., van Meer, G., and Sprong, H. (2007). Pre- and post-Golgi translocation of glucosylceramide in glycosphingolipid synthesis. J. Cell Biol. *179*, 101–115.

Hartfield, E.M., Fernandes, H.J.R., Vowles, J., Cowley, S.A., and Wade-Martins, R. (2012). Cellular reprogramming: a new approach to modelling Parkinson's disease. Biochem. Soc. Trans. *40*, 1152–1157.

Hartfield, E.M., Yamasaki-Mann, M., Ribeiro Fernandes, H.J., Vowles, J., James, W.S., Cowley, S.A., and Wade-Martins, R. (2014). Physiological characterisation of human IPS-derived dopaminergic neurons. PLoS One *9*, e87388.

Hoepken, H.-H., Gispert, S., Azizov, M., Klinkenberg, M., Ricciardi, F., Kurz, A., Morales-Gordo, B., Bonin, M., Riess, O., Gasser, T., et al. (2008). Parkinson patient fibroblasts show increased alpha-synuclein expression. Exp. Neurol. *212*, 307–313.

Høyer-Hansen, M., and Jäättelä, M. (2007). Connecting endoplasmic reticulum stress to autophagy by unfolded protein response and calcium. Cell Death Differ. *14*, 1576–1582.

Jang, A., Lee, H.J., Suk, J.E., Jung, J.W., Kim, K.P., and Lee, S.J. (2010). Non-classical exocytosis of alpha-synuclein is sensitive to folding states and promoted under stress conditions. J. Neurochem. *113*, 1263–1274.

Juhasz, G. (2012). Interpretation of bafilomycin, pH neutralizing or protease inhibitor treatments in autophagic flux experiments: novel considerations. Autophagy *8*, 1875–1876.

Kiskinis, E., and Eggan, K. (2010). Progress toward the clinical application of patient-specific pluripotent stem cells. J. Clin. Invest. *120*, 51–59.

Klionsky, D.J., Abdalla, F.C., Abeliovich, H., Abraham, R.T., Acevedo-Arozena, A., Adeli, K., Agholme, L., Agnello, M., Agostinis, P., Aguirre-Ghiso, J.A., et al. (2012). Guidelines for the use and interpretation of assays for monitoring autophagy. Autophagy *8*, 445–544.

Klucken, J., Poehler, A.-M., Ebrahimi-Fakhari, D., Schneider, J., Nuber, S., Rockenstein, E., Schlötzer-Schrehardt, U., Hyman, B.T., McLean, P.J., Masliah, E., et al. (2012). Alpha-synuclein aggregation involves a bafilomycin A 1-sensitive autophagy pathway. Autophagy *8*, 754–766.

Koga, H., Kaushik, S., and Cuervo, A.M. (2010). Altered lipid content inhibits autophagic vesicular fusion. FASEB J. *24*, 3052–3065.

Lee, H.J., Patel, S., and Lee, S.J. (2005). Intravesicular localization and exocytosis of alpha-synuclein and its aggregates. J. Neurosci. *25*, 6016–6024.

Lee, H.-J., Cho, E.-D., Lee, K.W., Kim, J.-H., Cho, S.-G., and Lee, S.-J. (2013). Autophagic failure promotes the exocytosis and intercellular transfer of α-synuclein. Exp. Mol. Med. *45*, e22.

Mazzulli, J.R., Xu, Y.H., Sun, Y., Knight, A.L., McLean, P.J., Caldwell, G.A., Sidransky, E., Grabowski, G.A., and Krainc, D. (2011). Gaucher disease glucocerebrosidase and alpha-synuclein form a bidirectional pathogenic loop in synucleinopathies. Cell *146*, 37–52.

McNeill, A., Duran, R., Hughes, D.A., Mehta, A., and Schapira, A.H. (2012). A clinical and family history study of Parkinson's disease in heterozygous glucocerebrosidase mutation carriers. J. Neurol. Neurosurg. Psychiatry *83*, 853–854.

McNeill, A., Magalhaes, J., Shen, C., Chau, K.-Y., Hughes, D., Mehta, A., Foltynie, T., Cooper, J.M., Abramov, A.Y., Gegg, M., et al. (2014). Ambroxol improves lysosomal biochemistry in glucocerebrosidase mutation-linked Parkinson disease cells. Brain *137*, 1481–1495.

Müller, F.-J., Schuldt, B.M., Williams, R., Mason, D., Altun, G., Papapetrou, E.P., Danner, S., Goldmann, J.E., Herbst, A., Schmidt, N.O., et al. (2011). A bioinformatic assay for pluripotency in human cells. Nat. Methods *8*, 315–317.

Nalls, M.A., Duran, R., Lopez, G., Kurzawa-Akanbi, M., McKeith, I.G., Chinnery, P.F., Morris, C.M., Theuns, J., Crosiers, D., Cras, P., et al. (2013). A multicenter study of glucocerebrosidase mutations in dementia with Lewy bodies. JAMA Neurol. *70*, 727–735.

Paillusson, S., Clairembault, T., Biraud, M., Neunlist, M., and Derkinderen, P. (2013). Activity-dependent secretion of alpha-synuclein by enteric neurons. J. Neurochem. *125*, 512–517.

Reczek, D., Schwake, M., Schröder, J., Hughes, H., Blanz, J., Jin, X., Brondyk, W., Van Patten, S., Edmunds, T., and Saftig, P. (2007). LIMP-2 is a receptor for lysosomal mannose-6-phosphate-independent targeting of beta-glucocerebrosidase. Cell *131*, 770–783.

Sardi, S.P., Clarke, J., Kinnecom, C., Tamsett, T.J., Li, L., Stanek, L.M., Passini, M.A., Grabowski, G.A., Schlossmacher, M.G., Sidman, R.L., et al. (2011). CNS expression of glucocerebrosidase corrects alpha-synuclein pathology and memory in a mouse model of Gaucher-related synucleinopathy. Proc. Natl. Acad. Sci. USA *108*, 12101–12106.

Schöndorf, D.C., Aureli, M., McAllister, F.E., Hindley, C.J., Mayer, F., Schmid, B., Sardi, S.P., Valsecchi, M., Hoffmann, S., Schwarz, L.K., et al. (2014). iPSC-derived neurons from GBA1-associated Parkinson's disease patients show autophagic defects and impaired calcium homeostasis. Nat. Commun. *5*, 4028.

Sevlever, D., Jiang, P., and Yen, S.-H.C. (2008). Cathepsin D is the main lysosomal enzyme involved in the degradation of alpha-synuclein and generation of its carboxy-terminally truncated species. Biochemistry *47*, 9678–9687.

Sidransky, E., Nalls, M.A., Aasly, J.O., Aharon-Peretz, J., Annesi, G., Barbosa, E.R., Bar-Shira, A., Berg, D., Bras, J., Brice, A., et al. (2009). Multicenter analysis of glucocerebrosidase mutations in Parkinson's disease. New Engl. J. Med. *361*, 1651–1661.

Tofaris, G.K. (2012). Lysosome-dependent pathways as a unifying theme in Parkinson's disease. Movement Disord. *27*, 1364–1369.

Tomlinson, P.R., Zheng, Y., Fischer, R., Heidasch, R., Gardiner, C., Evetts, S., Hu, M., Wade-Martins, R., Turner, M.R., Morris, J., et al. (2015). Identification of distinct circulating exosomes in Parkinson's disease. Ann. Clin. Transl. Neurol. *2*, 353–361.

van Wilgenburg, B., Browne, C., Vowles, J., and Cowley, S.A. (2013). Efficient, long term production of monocyte-derived macrophages from human pluripotent stem cells under partly-defined and fully-defined conditions. PLoS One *8*, e71098.

Vitner, E.B., Dekel, H., Zigdon, H., Shachar, T., Farfel-Becker, T., Eilam, R., Karlsson, S., and Futerman, A.H. (2010). Altered expression and distribution of cathepsins in neuronopathic forms of Gaucher disease and in other sphingolipidoses. Hum. Mol. Genet. *19*, 3583–3590.

Wei, R.R., Hughes, H., Boucher, S., Bird, J.J., Guziewicz, N., Van Patten, S.M., Qiu, H., Pan, C.Q., and Edmunds, T. (2011). X-ray and biochemical analysis of N370S mutant human acid β-glucosidase. J. Biol. Chem. *286*, 299–308.

Yorimitsu, T., Nair, U., Yang, Z., and Klionsky, D.J. (2006). Endoplasmic reticulum stress triggers autophagy. J. Biol. Chem. *281*, 30299–30304.

Stem Cell Reports, Volume *6*

Supplemental Information

ER Stress and Autophagic Perturbations Lead to Elevated Extracellular α-Synuclein in *GBA-N370S* Parkinson's iPSC-Derived Dopamine Neurons

Hugo J.R. Fernandes, Elizabeth M. Hartfield, Helen C. Christian, Evangelia Emmanoulidou, Ying Zheng, Heather Booth, Helle Bogetofte, Charmaine Lang, Brent J. Ryan, S. Pablo Sardi, Jennifer Badger, Jane Vowles, Samuel Evetts, George K. Tofaris, Kostas Vekrellis, Kevin Talbot, Michele T. Hu, William James, Sally A. Cowley, and Richard Wade-Martins

Figure-S1 (Wade-Martins)

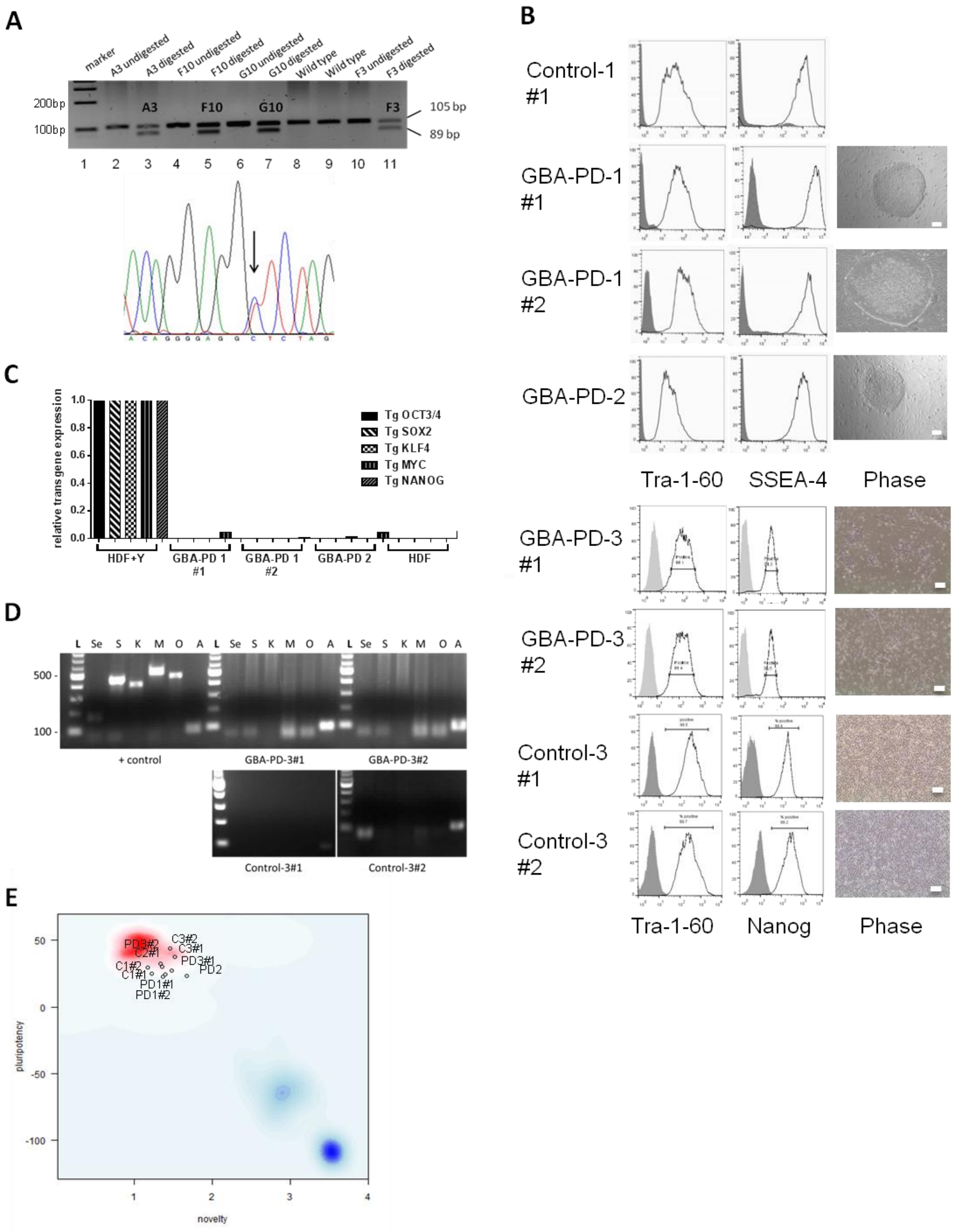

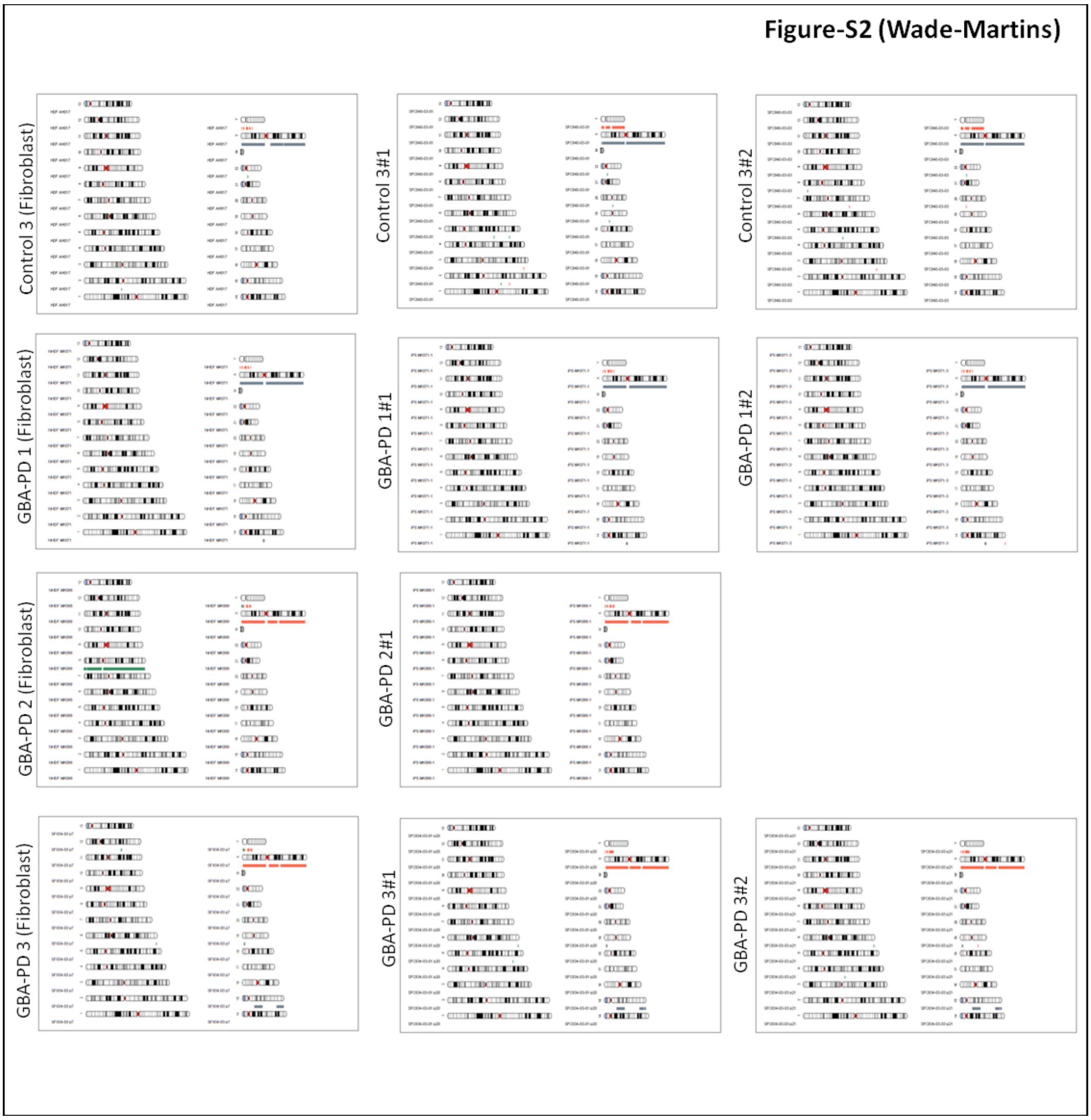
Figure-S2 (Wade-Martins)
Control 3 (Fibroblast)
Control 3#1
Control 3#2
GBA-PD 1 (Fibroblast)
GBA-PD 1#1
GBA-PD 1#2
GBA-PD 2 (Fibroblast)
GBA-PD 2#1
GBA-PD 3 (Fibroblast)
GBA-PD 3#1
GBA-PD 3#2

Figure-S3 (Wade-Martins)

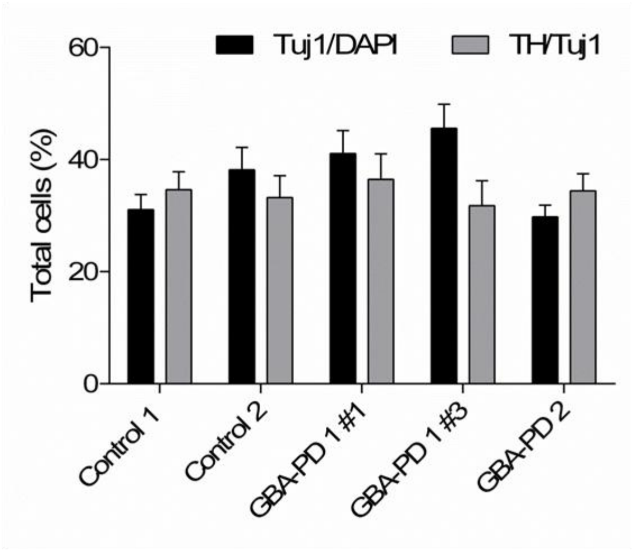

Figure-S4 (Wade-Martins)

A

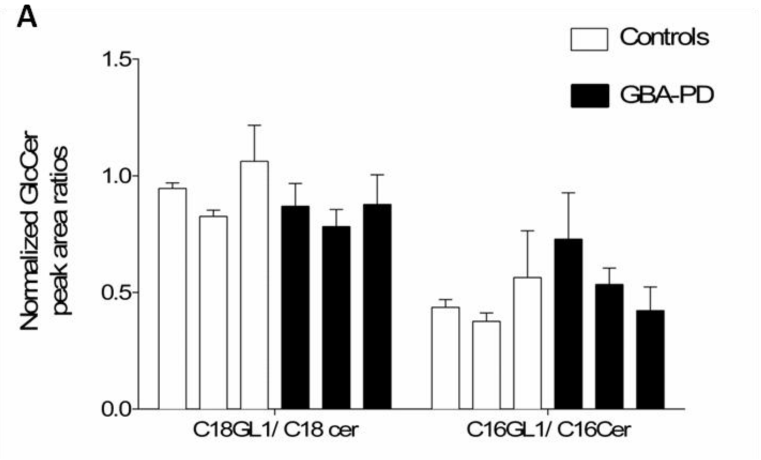

B

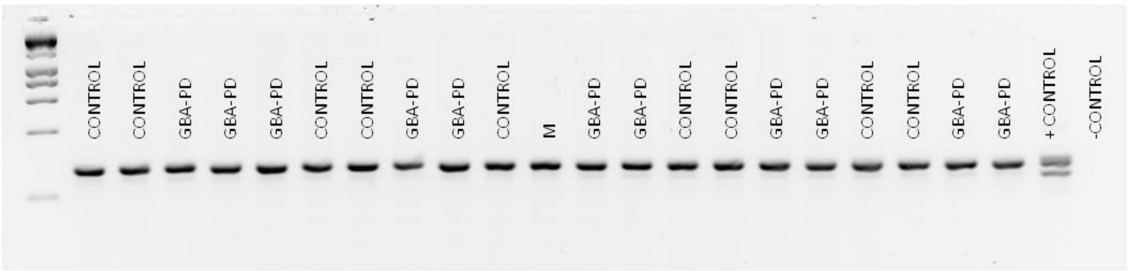

Figure-S5 (Wade-Martins)

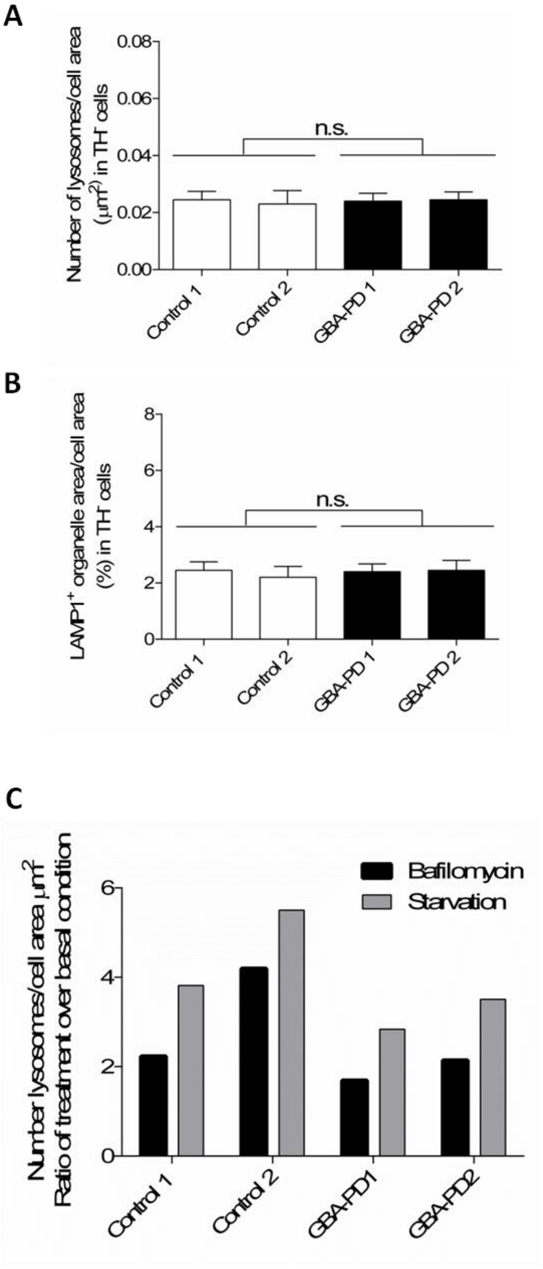

Figure-S6 (Wade-Martins)

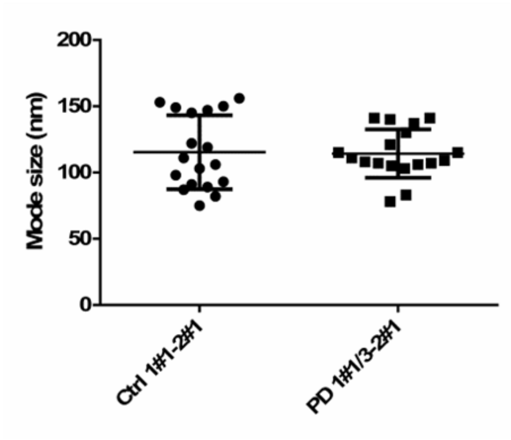

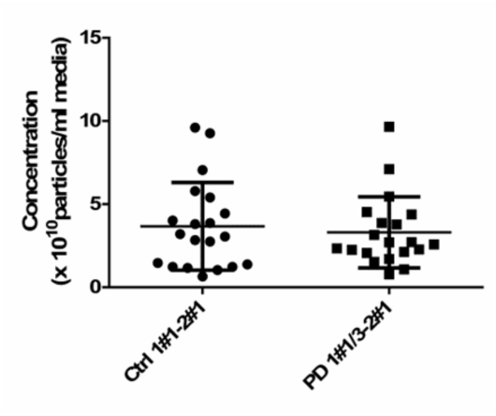

Figure-S7 (Wade-Martins)

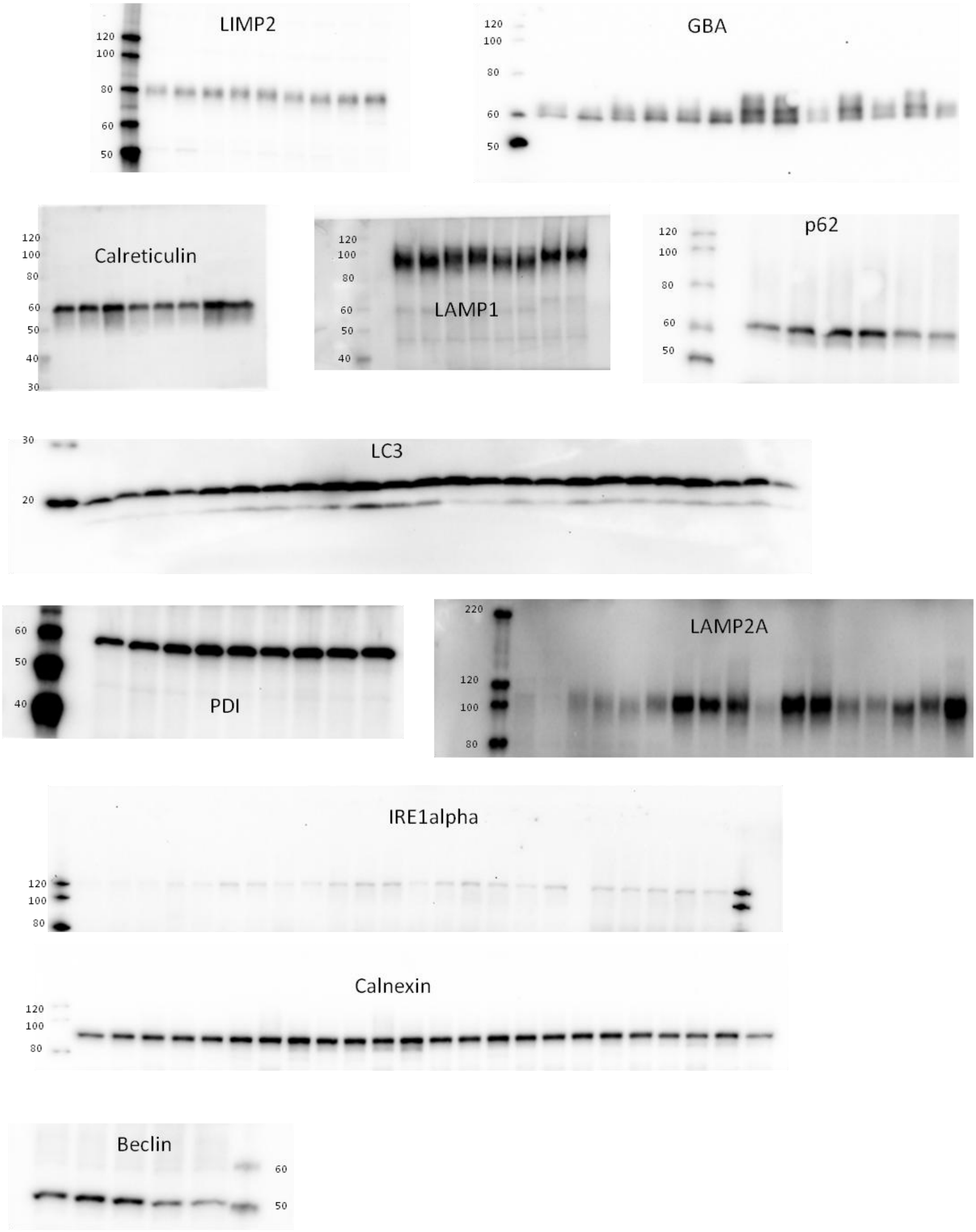

Supplemental Figures

Figure S1. Related to Figure 1. Establishment of PD iPSC lines from patient fibroblasts. (A) *GBA* N370S mutation screening. Novel XhoI restriction site identified positive cases by PCR which were then confirmed by genomic sequencing analysis – arrow indicates mutation site. (B) PD iPSC lines express expected pluripotency proteins, as shown by FACs for Tra-1-60, SSEA-4 and Nanog (grown feeder-free); open black plot represents antibody, filled grey plot represents isotype control; Right-hand panel shows the expected iPSC colony morphology, with high nucleus to cytoplasm ratio by phase-contrast microscopy; cells are densely packed when photographed on feeders, but SFC lines are photographed one day after thawing onto matrigel, so have not yet clustered tightly together. Scale bar = 100 µm. iPS-NHDF-1 (previously published) are included as a positive control reference cell line for FACs. (C) Transgene silencing in iPSC lines. qRT-PCR for each transgene, normalised to actin endogenous control, and expressed in comparison to the expression of transgenes from fibroblasts 5 days post-infection with the Yamanaka reprogramming retroviruses (HDF+Y). Uninfected fibroblasts (HDF) serve as a negative control. (D) Cytotune Sendai virus clearance in iPSC lines by RT-PCR. L, Log2 ladder; Se, Sendai backbone 181 bp; S, Sox2 451 bp; K, Klf4 410 bp; M, c-myc 532 bp; O, Oct-4 483 bp; A, β-actin control 92 bp; + control, fibroblasts infected with Cytotune 5 days previously. iPSc lines show the correct size band for β-actin, and no bands corresponding to the reprogramming virus PCR product sizes. (E) PluriTest analysis of Illumina HT12v4 transcriptome array data shows the tested PD iPSC lines cluster with the (previously published) control iPSC lines pluripotent stem cells in the red cloud and not with differentiated cells (blue clouds). Each circle represents one iPSC line.

Figure S2. Related to Figure 1. Karyotype analysis of iPSC lines used in the study. Genome integrity was assessed by Illumina Human CytoSNP-12v2.1 or OmniExpress24 SNP array and karyograms produced using KaryoStudio software (Illumina). Amplifications (green), deletions (orange) and LOH regions (grey) are shown alongside the relevant chromosome (except that in females the X chromosomes are annotated with grey, and single-copy sex chromosomes are annotated orange).

Figure S3. Related to Figure 1. Similar differentiation efficiency across lines. Neuronal and dopaminergic differentiation efficiency for control and heterozygous *GBA-N370S* mutant PD patient lines. Approximately 40% of cells developed into Tuj1-positive neurons, approximately 35% of which were Tuj1/TH double-positive. Data represent mean ± SEM of at least 3 independent differentiations. (Two-way ANOVA with Tukey post hoc analysis, $P>0.05$, not significant).

Figure S4. Related to Figures 2 and 3. (A) No accumulation of GlcCer substrate as measured by mass spectrometry quantification of individual GlcCer species normalized to the ceramide precursor. Each bar represents mean ± SEM of differentiated lines from different individuals done in triplicate (n=3). (B) No activation of XBP1 mRNA splicing events for dopaminergic neuronal cultures. After dopaminergic neuronal differentiation, mRNA was extracted and screened for XBP1 splicing events by RT-PCR for controls and heterozygous *GBA-N370S* cultures.

Figure S5. Related to Figure 6. Analysis of the lysosomal compartment. (A) No alteration for the number of lysosomes in non-TH cells in heterozygous *GBA-N370S* dopaminergic cultures when compared to controls as determined by EM analysis. (B) No differences found for the size of lysosomes in non-TH cells in heterozygous *GBA-N370S* dopaminergic cultures when compared to controls as determined by EM analysis. (C) Efficient lysosomal biogenesis in dopaminergic neurons determined by EM. 24 h starvation or 6 h bafilomycin treatment resulted in an increased number of lysosomes in TH positive neurons relatively to basal condition for both control and *GBA-N370S* dopaminergic cultures, as determined by EM quantification of LAMP1 labeled structures.

Figure S6. Related to Figure 7. Microvesicle analysis of culture media. For the microvesicle analysis of culture media no differences were observed for the size (D) or concentration (E) of exosomes between control and heterozygous GBA-N370S groups.

Figure S7. Representative western blots for antibodies used. Multiple western blots are given together with the respective associated molecular weight markers, for the clarification of the bands of the multiple proteins quantified across the manuscript.

Supplemental Table

Table S1 Summary of PD patients derived iPSC lines used in the study.

ID	Diagnosis	Genotype	Age of Biopsy (years)	Age of onset (years)	Number of iPS clones generated	ID of iPS clones used	Original IDs	Characterization
Control-1	healthy	wt/wt	44	-	2	Ctrl 1#1<br>Ctrl 1#2	NHDF-1<br>NHDF-2	Hartfield *et al* 2014
Control-2	healthy	wt/wt	36	-	3	Ctrl 2#1	OX1-19	Van Wilgenburg *et al* 2013
Control-3	healthy	wt/wt	67	-	6	Ctrl 3#1<br>Ctrl 3#2	SFC840-03-01 [AH017-11]<br>SFC840-03-03 [AH017-13]	This study
GBA-PD-1	PD	N370S/wt	81	77	4	PD 1#1<br>PD 1#2	MK071-1<br>MK071-3	This study
GBA-PD-2	PD	N370S/wt	46	40	3	PD 2#1	MK088-1	This study
GBA-PD-3	PD	N370S/wt	72	69	3	PD 3#1<br>PD 3#2	SFC834-03-01<br>SFC834-03-03	This study

Supplemental Experimental Procedures:

Participant recruitment

Participants were recruited to this study having given signed informed consent, which included mutation screening and derivation of hiPSC lines from skin biopsies (Ethics committee: National Health Service, Health Research Authority, NRES Committee South Central – Berkshire, UK, who specifically approved this part of the study - REC 10/H0505/71). All the patients included in our study fulfilled UK Brain Bank diagnostic criteria for clinically probable PD at presentation (Hughes et al., 1992). *GBA*-associated parkinsonism is known to present and subsequently progress in a different way from patients with sporadic PD. In general, a younger age of onset, increased risk of early Parkinson's-related dementia, early falls and postural imbalance and initial dementia phenotype are recognized with heterozygous GBA mutations (Asselta et al., 2014; Gan-Or et al., 2015; Winder-Rhodes et al., 2013). Patients 1 and 2 presented with akinetic-rigid parkinsonism, and maintained a good levodopa-response for their first 5 years of treatment without significant falls or dementia. Patient 3 presented with akinetic-rigid parkinsonism, failed to respond to dopaminergic medication and subsequently rapidly progressed more quickly with early dementia and frequent falls two years later.

***GBA*-N370S mutation screening**

Study participants were screened for the N370S mutation in the GBA gene by extracting genomic DNA from blood samples using the AutoPure LS® Kit (QIAGEN). PCR reactions were carried out using AmpliTaq Gold DNA polymerase (Applied Biosystems). Primer sequences were used as follows: 5'-GCCTTTGTCCTTACCCTC*G -3' and 5'- GACAAAGTTACGCACCCAA-3'. * indicates a mismatch that was engineered into the forward primer in order to create a XhoI restriction site in the PCR product from participants carrying the N370S mutation as described previously(Aharon-Peretz et al., 2004). The resulting PCR product was digested with XhoI (NEB) and resolved by agarose gel electrophoresis. Mutations were confirmed by sequencing. Briefly, DNA was treated with an ExoSAP reaction as follows: 1X SAP buffer, shrimp alkaline phosphatase (500 U; SAP, Promega), Exonuclease I (2 U; NEB). Samples were incubated at 37 °C for 1 hour and then at 80 °C for 20 minutes. The sequencing reaction was performed according to BigDye Terminator v3.1 Cycle Sequencing protocol (Applied Biosystems). Following a clean up step, the sequencing read was performed on a 3700 DNA Analyser (Applied Biosystems) sequencing platform.

Culture and reprogramming of primary fibroblasts

All iPSC lines used in this study were derived from skin biopsies and reprogrammed using the same methodology and in the same laboratory. Skin punch biopsies (4 mm in diameter) were obtained from participants and the subcutaneous tissue removed. The dermal sample was cut into several small pieces and cultured in ADMEM (Invitrogen) supplemented with USDA-approved foetal bovine serum (10%; Sigma) and penicillin/streptomycin (1%) in a humidified incubator (37 °C, 5% CO_2). Glass coverslips were placed over the biopsy to facilitate adhesion. Fibroblast were reprogrammed at passage 3-5, using either a) reprogramming retroviruses carrying individual reprogramming genes (pMXs plasmids encoding the reprogramming genes were obtained from Addgene -17220: pMXs-hc-MYC, 17219: pMXs-hKLF4, 17218: pMXs-hSOX2, 17217: pMXs-hOCT3/4, 13354: pMXs-Nanog) as in the original papers by Takahashi and Yamanaka(Kitamura et al., 2003; Takahashi et al., 2007; Takahashi and Yamanaka, 2006). They were packaged using the Plat-GP retroviral packaging cell line (containing retroviral gag and pol genes) and cotransfected with VSV-G, using PEI, to make iPS retrovirus vectors. Reprogramming was carried out following[4]; Specifically, 50,000 fibroblasts were infected on days 0 and 1 with an equal volume of each virus supernatant except Oct which was used at a 3-fold dose, plus 5 µg/ml polybrene and spinoculation (1200 g for 45 minutes at 16°C). Or b) One control donor (SFC840) and one GBA donor (SFC834) was reprogrammed using Cytotune Sendai virus reprogramming kit (Invitrogen) according to the manufacturer's instructions, scaled down to infect 50,000 fibroblasts at the same recommended MOI. For both reprogramming methods, fibroblasts were transferred onto mitotically-inactivated 'Pathology Oxford' (Gardner, 1982) outbred mouse embryonic feeder cells (MEF) on 0.1% gelatin coated plates (Sigma) on day 4, and from day 5 onwards were cultured in standard KnockOut serum replacement medium (Knock-out DMEM (Invitrogen), KO-Serum Replacement (20% Invitrogen), Glutamax-I (2 mM Invitrogen), non-essential amino acids (1%, Invitrogen), penicillin (100 U/mL Invitrogen), streptomycin (100 µg/mL Invitrogen), 2-ME (55 µM Invitrogen) and bFGF (10 ng/mL R&D)) (supplemented with 50 µg/ml ascorbic acid and 0.5 µM Valproic acid (both from Sigma) for the retroviral protocol to enhance reprogramming efficiency),

replacing 50% medium on alternate days, and substituting with MEF-conditioned medium from day 10 onwards. Colonies displaying iPSC morphology were picked on day ~28 and passaged on MEFs by manual dissection every 5-7 days. iPSC lines were adapted to feeder-free culture conditions by three passages on Matrigel coated plates (BD Matrigel hESC-qualified Matrix) in mTeSR™1 (StemCell Technologies), using 0.5 mM EDTA in PBS to dissociate cells to small clumps (Beers et al., 2012). For some applications, enzymatic dissociation (TryplE Express, Gibco) supplemented with Rock inhibitor Y27632 (10 µM; Calbiochem) on the day of passage was used. – in this case, the number of feeder-free passages was always kept to an absolute minimum to reduce the likelihood of genetic change. Cells were frozen in SNP-QCed batches of at least 30 vials (within a narrow window of passage numbers, ~p15-30), from which cells would be thawed for each experiment, to ensure consistency across experiments.

Characterisation of iPSCs

Morphological assessment, passageability and immunocytochemistry for markers of pluripotency were carried out as initial indicators of pluripotency. After converting hiPSCs to feeder-free culture on Matrigel (BD Biosciences) for at least 3 passages, cells were harvested for more in depth characterisation analyses and RNA and genomic DNA was made using an All-Prep kit (Qiagen).

For fluorescence activated cell sorting (FACs) of pluripotency markers the following antibodies were used, with appropriate isotype control, at the same concentration, from the same supplier (clone, isotype control, supplier): TRA-1-60 (B119983, IgM-488, Biolegend), SSEA-4-633 (MAB1435, IgG3-488, R&D), on fresh cells, or, for staining in combination with Nanog (2985S, IgG-647, Cell Signaling), they were fixed for 10 minutes in 2% paraformaldehyde in PBS (Alfa Aesar), permeabilised in 100% methanol at -20 for at least 30 mins before staining,. Fluorescence was measured using a FACS Calibur (Becton Dickinson), and data was analysed using FlowJo software.

qRT-PCR for assessing the degree of silencing of retroviral transgene sequences was carried out using primer sequences published by Takahashi et al. supplementary table S12[4], except that in place of the published reverse primer (pMXs-AS3200 TTA TCG TCG ACC ACT GTG CTG CTG), we used our own designed primer, pMXs-AS3200v2 (TTA TCG TCG ACC ACT GTG CTG GCG) which had exactly the same sequence as the target pMXs vector backbone sequence and which therefore amplified more efficiently; also, for amplifying transgene mNanog, the forward primer GCT CCA TAA CTT CGG GGA GG. RNA was reverse transcribed using a RetroScript kit (Ambion), using 2ug template RNA in 20 µl reaction volume. 2 µl of 1:10 dilution of cDNA product was used in a 25 µl qRT-PCR reaction. QRT-PCR was carried out on an Applied Biosystems StepOne Plus Real Time PCR machine, with StepOne software, using Applied Biosystems 2xSYBR green PCR mix + ROX and 60°C anneal, Target gene transcript levels were compared to actin B control (actin B primers, Eurogentec), and subsequently to fibroblasts harvested 5 days after infection with the reprogramming vectors.

RT-PCR to assess clearance of Cytotune Sendai virus-delivered reprogramming genes was preformed according to the manufacturer's instructions and run on a 1.5% agarose gel with Log2 ladder (NEB). Positive controls (fibroblasts infected 5 days previously) were always run in parallel. Primers were SeV F: GGATCACTAGGTGATATCGAGC, R: ACCAGACAAGAGTTTAAGAGATATGTATC 181bp; SOX2 F: ATGCACCGCTACGACGTGAGCGC, R: AATGTATCGAAGGTGCTCAA 451bp; KLF4 F: TTCCTGCATGCCAGAGGAGCCC, R: AATGTATCGAAGGTGCTCAA 410bp; c-MYC F: TAACTGACTAGCAGGCTTGTCG, R: TCCACATACAGTCCTGGATGATGATG 532bp; OCT4 F: CCCGAAAGAGAAAGCGAACCAG, R: AATGTATCGAAGGTGCTCAA 483bp; β-Actin control Eurogentec 92bp

Genome integrity was assessed by an Illumina Human CytoSNP-12v2.1 beadchip array (~300,000 markers) or OmniExpress24 array (700,000 markers, SFC840 and SFC834 lines) and analyzed using KaryoStudio and GenomeStudio software (Illumina), comparing the iPSC lines to the parental fibroblasts. Note that one of the parental fibroblast lines was a mosaic (a proportion of GBA-PD-2 cells having trisomy 8), but the derived iPSc line used in this study was a normal karyotype. Clinical records indicated that there was no known close relatedness between the individuals used in this study.

Analysis of pluripotency was performed on RNA extracted from iPSC lines using the Illumina HT12v4 transcriptome array. The image data files were then uploaded to www.pluritest.org and scored for pluripotency, as previously described (Müller et al., 2011). According to this analysis, pluripotent cell lines will cluster in the top left quadrant of the graph, indicating high pluripotency score and low novelty score (novelty meaning genes

that are not commonly expressed in iPSCs). All lines tested had acceptable pluripotency scores, but note that one line (iPS-MK088-1) appeared by this assessment to express more 'novelty genes' than the other lines – however, this was not reflected in its passageability, or the other assays undertaken, nor in the ability of the lines to differentiate to Embryoid Bodies (EBs) and neuronal lineages.

Differentiation of DA neurons

Prior to differentiation, iPSC lines were adapted to feeder-free conditions using Matrigel (BD). mTeSR™1 (StemCell Technologies) was supplemented with Y27632 (10μM; Calbiochem) on the day of passage. Embryoid bodies (EBs) were formed by dissociation of iPSCs with TryplE and seeded into Aggrewell plates (10,000 cells per EB; Stem Cell Technologies) in mTeSR™-1 medium supplemented with Y27632 (10 μM), with a 75% daily medium change. After 4 days, neural induction was initiated.

All materials for differentiation were obtained from Life Technologies unless otherwise stated. EBs were plated onto Geltrex-coated plates in Neural Induction medium 1 (DMEM/F12 supplemented with L-glutamine [2 mM], N2 supplement, bovine serum albumin [1 mg/ml], Y27632 [10 μM; Tocris], SB431542 [10 μM, Tocris], noggin [200 ng/ml], sonic hedgehog [500 ng/ml; SHH C24II; R&D Systems], CHIR99021 [0.7 μM; Stemgent] and antibiotic/ antimycotic [1% v/v]). After 12 days, medium was changed to Neural Induction medium 2 (as NI1, without SB431542 and noggin, SHH C24II [20 ng/ml], FGF8a [100 ng/ml; R&D Systems], heparin [5 μg/ml; Sigma], BDNF [20 ng/ml] and ascorbic acid [200 μM; Sigma]) and incubated for 8 days, until the appearance of dense neural rosette structures became apparent. Neural progenitor cells were manually selected and replated onto poly-D-lysine/laminin-coated plates in final differentiation medium (DMEM/F12 supplemented with L-glutamine [2 mM], N2 supplement, BDNF [20 μg/ml], glial-derived neurotrophic factor [GDNF, 20 μg/ml], N^6,2′-O-dibutyryladenosine 3′,5′-cyclic monophosphate sodium salt [dCAMP, 0.5 mM; Sigma], laminin [1 μg/ml] and antibiotic/antimycotic (1% [v/v]). Neurons were matured for 2-6 weeks in this medium before experimental procedures were carried out. For additional experiments (Figures 2E, 7E and S4), cells were differentiated as previously described (Kriks et al., 2011)

Immunocytochemistry

Cells were fixed in 4% paraformaldehyde and permeabilised in 0.1% Triton-X100 prior to immunostaining. Coverslips were blocked in 10% goat or donkey serum for 1 hour before incubating with primary antibodies overnight at 4°C. Antibodies used as follows: FoxA2 (1:500; R&D systems), TUJ1 (β-3 tubulin) (1:500; Covance), TH (1:500; Millipore), LC3 (1:200; Nanotools), PiTX3 (1:100, Life Technologies) and Nurr1 (1:400, Millipore). Secondary antibodies (Alexa fluor, Life Technologies) were incubated for 1 hour at room temperature before mounting and analysis. Images were captured using a Leica SP5 confocal or a EVOS FL microscope.

RT-PCR analysis

RNA was extracted from cells using Trizol (Life Technologies) and purified using the RNeasy kit (QIAGEN). Reverse transcription was performed using Superscript III (Life Technologies) according to manufacturer's instructions. Polymerase chain reactions (PCR) were set up with 20 ng of cDNA using GoTaq DNA Polymerase products (Promega). Primer sequences used as follows: FOXA2: 5'-GACAAGTGAGAGAGCAAGTG-3' and 5'-ACAGTAGTGGAAACCGGAG-3'; LMX1A: 5'-AACGACAGCTTCTGGCATGA-3' and 5'-TCAAGATGGTTCTCGGACGT-3'; EN1: 5'-GCTATCCTACTTATGGGCTCA-3' and 5'-GGAGTGGTTGTACAGTCCCT-3'; NURR1: 5'-CGACATTTCTGCCTTCTCC-3' and 5'-GGTAAAGTGTCCAGGAAAAG-3'; OCT4: 5'-AAAGCTCTGCAGAAAGAACTCG-3' and 5'-CTCACTCGGTTCTCGATACTGG-3'; GAPDH: 5'-CAGGGCTGCTTTTAACTCTGG-3' and 5'-AAGTTGTCATGGATGACCTTGG-3'.

Western blot

Western blotting was carried out on whole cell lysates extracted using RIPA buffer (Tris [50 mM, pH 8], sodium chloride [150 mM], sodium dodecyl sulphate [SDS; 0.1% w/v], sodium deoxycholate [0.5 % w/v] and nonidet-P40 [1% w/v]). Before loading, samples were denatured for 5 minutes at 100 °C. Protein separation was achieved using SDS polyacrylamide gel electrophoresis and transferred onto PVDF membrane. Antibodies used as follows: TH (1:500; Millipore, AB1542), GBA (1:200; Abcam, ab55080), β-actin (1:10,000; Abcam, ab8227), LIMP2 (1:1000; ProSci, 4621), BiP (1:200; Abcam, ab21685), LC3 (1:200; Nanotools, 5F10), Beclin-1 (1:500; Abcam, ab51031), α-synuclein (1:200; Covance, SIG-39730-200), Cathepsin D (1:500; Abcam, ab6313), P62 (1:500; Abcam, ab56416).

Immunogold electron microscopy

Cells grown on polyester filters were fixed in 3% paraformaldehyde/0.05% glutaraldehyde and prepared for immunogold EM by standard methods (Morris et al., 2006). Filters were stained with uranyl acetate (2 % w/v in distilled water), dehydrated through increasing concentrations of methanol (70-100 %) and embedded in LR Gold resin (Agar, Reading UK). Ultra-thin sections (50-80 nm) were prepared by use of a Reichert Ultracut S ultratome (Leica, Milton Keynes, UK), mounted on 200-mesh nickel grids, incubated at room temperature with either anti-profilin antibody (dilution 1:200, 2 hour) or anti-TH antibody (1:200, 2 hour) followed by Protein A-15 nm gold complex for 1 hour (1:60). All antisera were diluted in 0.1M phosphate buffer containing 0.1 % egg albumin. As a negative control, the primary antibody was replaced by non-immune sera and immunogold labelling was not observed. After immunolabelling sections were lightly counterstained with lead citrate and uranyl acetate and examined with a JEOL transmission electron microscope (JEM-1010, JEOL, Peabody, MA, USA) and representative micrographs were prepared. The area of the cells was analysed using Axiovison (version 4.5) image analysis software and the number of lysosomes counted for 8 cells per group**.** Lysosomes were manually counted by an investigator blinded to genotype and treatment. All blocks were coded, labeled, and quantified prior to counting and analysis. Decoding was only done when all analysis was complete.

GCase activity assay

GCase activity was measured as described previously. Briefly neurons were lysed and sonicated in GCase lysis buffer (citrate phosphate buffer supplemented with 0.25 % (v/v) Triton-X100 and 0.25% (w/v) taurocholic acid pH 5.4). Following incubation on ice for 30 minutes, samples were centrifuged for 10 minutes at 4°C. The supernatant was incubated with 4-methylumbelliferyl β-D-glucopyranosidase (5 mM) and incubated at 37°C for 1 hour. The reaction was stopped by adding excess glycine phosphate buffer (0.2 M, pH 10.4) and resulting fluorescence detected on a Synergy HT plate reader (BioTek). Excitation at 360 nm and emission 440 nm was used. Samples treated with conduritol B epoxide (2.5 mM; Enzo Life Sciences) were used as a negative control and to provide background values for GCase activity.

Exosome isolation

Conditioned media from iPSC-derived dopaminergic neuronal cultures were subjected to serial centrifugation at 1,500 g for 10 min followed by 17,000 g for 30 min with all steps performed at 4 °C. The resulting supernatant was filtered using a 0.2 μm filter and spun at 160,000 g for 1 h using a MLS-50 swinging rotor. The pellet from each group was resuspended in Hanks Balanced Salt Solution and aliquoted for Nanoparticle Tracking Analysis, and immunoblotting. For immunoblotting, 2.2x109 microvesicles were loaded per condition, which would correspond to microvesicles extracted from about 12 ml of conditioned medium from each patient's iPSC line.

Nanoparticle Tracking Analysis

Nanoparticle Tracking Analysis (NTA) uses light scattering to enable direct, real-time visualisation and analysis of nanoparticles and uses the rate of Brownian motion to estimate individual particle size. NTA is a useful technique for making rapid size and concentration measurements of microvesicle/exosome preparations because requirements of sample preparation are simple. This is an established method to measure size and concentration in the exosome field (Gardiner et al., 2013). Exosome size and concentration were assessed using a NS500

instrument (Nanosight Ltd. Amesbury, UK) equipped with a 405 nm laser and a CMOS camera. Samples were diluted in filtered PBS immediately prior to use. 5 x 30 second videos were recorded for each sample (camera gain 350; shutter speed 14.99 ms). Videos were analysed using NTA software (version 2.3). Instrument calibration was verified by analyzing silica microspheres (Polysciences, Warrington, PA) prior to each analysis.

α -syn ELISA

Briefly, each ELISA plate (Corning Costar) was coated for 24 hrs at room temperature with 0.5 µg/ml of Syn-1 antibody (50 µl per well) in 100 mM NaHCO3, pH 9.3. The plates were washed three times in wash buffer (50 mM Tris-HCl, 150 mM NaCl and 0.04% Tween-20) and 50 µl of sample was added. Recombinant human α-Syn (Chemicon) (as standard) was diluted in 50% TBST/BSA (10 mM Tris-Cl, pH 7.6, 100 mM NaCl, 0.1% Tween-20 and 1% BSA) and 50% cell culture medium. Samples of conditioned medium were 2-fold diluted in TBST/BSA prior to addition to the wells. To allow antigen binding, plates were incubated at 37 ^{o}C for 2.5 hrs. After washing three times with wash buffer, 50 µl of HRP-conjugated C-20 antibody (1:4000 diluted in TBST/BSA) was added to each well and further incubated for 1 hr at room temperature. The wells were washed and 50 µl of chemiluminogenic HRP substrate (UptiLight HS ELISA HRP substrate, Interchim) was added to each well. Following incubation for 10 minutes at room temperature, chemiluminescence was integrated for 1s. Standards and conditioned medium samples were measured at least in duplicate.

α -syn Meso Scale Discovery (MSD)

Conditioned media samples (100 µl) from iPSC-derived dopaminergic neuronal cultures were loaded on commercial available human α–synuclein plates purchased from Meso Scale Discovery (Rockville, MD, USA, Cat# K151TGD-2). Assay was performed following the manufacturer's instructions and read using a MESO QuickPlex SQ 120 instrument (Meso Scale Discovery).

Glucosylceramide (GlcCer) and ceramide (Cer) quantification.

Quantitative analysis of sphingolipids was performed using liquid chromatography and tandem mass spectrometry as described previously (Gegg et al., 2015). Briefly, cell pellets were extracted with 0.5 mL of a solution of acetonitrile:methanol:water (97:2:1, v/v/v). Samples were analyzed with an AB Sciex API-5000 mass spectrometer (AB Sciex, Framingham, MA).

Statistics

Results are expressed as mean ± SEM from performing at least three independent differentiation experiments per line each analysed in triplicate except otherwise stated. Statistical significance was determined by Student's *t*-test or with two-way ANOVA.

Supplemental References:

Aharon-Peretz, J., Rosenbaum, H., and Gershoni-Baruch, R. (2004). Mutations in the glucocerebrosidase gene and Parkinson's disease in Ashkenazi Jews. The New England journal of medicine *351*, 1972-1977.

Asselta, R., Rimoldi, V., Siri, C., Cilia, R., Guella, I., Tesei, S., Solda, G., Pezzoli, G., Duga, S., and Goldwurm, S. (2014). Glucocerebrosidase mutations in primary parkinsonism. Parkinsonism & related disorders *20*, 1215-1220.

Beers, J., Gulbranson, D., George, N., Siniscalchi, L., Jones, J., Thomson, J., and Chen, G. (2012). Passaging and colony expansion of human pluripotent stem cells by enzyme-free dissociation in chemically defined culture conditions. Nat Protocols *7*, 2029-2040.

Gan-Or, Z., Amshalom, I., Kilarski, L.L., Bar-Shira, A., Gana-Weisz, M., Mirelman, A., Marder, K., Bressman, S., Giladi, N., and Orr-Urtreger, A. (2015). Differential effects of severe vs mild GBA mutations on Parkinson disease. Neurology *84*, 880-887.

Gardiner, C., Ferreira, Y.J., Dragovic, R.A., Redman, C.W., and Sargent, I.L. (2013). Extracellular vesicle sizing and enumeration by nanoparticle tracking analysis. Journal of extracellular vesicles *2*.

Gardner, R.L. (1982). Investigation of cell lineage and differentiation in the extraembryonic endoderm of the mouse embryo. Journal of embryology and experimental morphology *68*, 175-198.

Gegg, M.E., Sweet, L., Wang, B.H., Shihabuddin, L.S., Sardi, S.P., and Schapira, A.H. (2015). No evidence for substrate accumulation in Parkinson brains with GBA mutations. Movement disorders : official journal of the Movement Disorder Society *30*, 1085-1089.

Hughes, A.J., Daniel, S.E., Kilford, L., and Lees, A.J. (1992). Accuracy of clinical diagnosis of idiopathic Parkinson's disease: a clinico-pathological study of 100 cases. Journal of neurology, neurosurgery, and psychiatry *55*, 181-184.

Kitamura, T., Koshino, Y., Shibata, F., Oki, T., Nakajima, H., Nosaka, T., and Kumagai, H. (2003). Retrovirus-mediated gene transfer and expression cloning: powerful tools in functional genomics. Experimental hematology *31*, 1007-1014.

Kriks, S., Shim, J.W., Piao, J., Ganat, Y.M., Wakeman, D.R., Xie, Z., Carrillo-Reid, L., Auyeung, G., Antonacci, C., Buch, A., *et al.* (2011). Dopamine neurons derived from human ES cells efficiently engraft in animal models of Parkinson's disease. Nature *480*, 547-551.

Morris, J.F., Omer, S., Davies, E., Wang, E., John, C., Afzal, T., Wain, S., Buckingham, J.C., Flower, R.J., and Christian, H.C. (2006). Lack of annexin 1 results in an increase in corticotroph number in male but not female mice. Journal of neuroendocrinology *18*, 835-846.

Müller, F.-J., Schuldt, B.M., Williams, R., Mason, D., Altun, G., Papapetrou, E.P., Danner, S., Goldmann, J.E., Herbst, A., Schmidt, N.O., *et al.* (2011). A bioinformatic assay for pluripotency in human cells. Nature methods *8*, 315-317.

Takahashi, K., Tanabe, K., Ohnuki, M., Narita, M., Ichisaka, T., Tomoda, K., and Yamanaka, S. (2007). Induction of Pluripotent Stem Cells from Adult Human Fibroblasts by Defined Factors. Cell *131*, 861-872.

Takahashi, K., and Yamanaka, S. (2006). Induction of pluripotent stem cells from mouse embryonic and adult fibroblast cultures by defined factors. Cell *126*, 663-676.

Winder-Rhodes, S.E., Evans, J.R., Ban, M., Mason, S.L., Williams-Gray, C.H., Foltynie, T., Duran, R., Mencacci, N.E., Sawcer, S.J., and Barker, R.A. (2013). Glucocerebrosidase

mutations influence the natural history of Parkinson's disease in a community-based incident cohort. Brain : a journal of neurology *136*, 392-399.
